# Bacteria‐Based Cancer Immunotherapy

**DOI:** 10.1002/advs.202003572

**Published:** 2021-02-10

**Authors:** Xuehui Huang, Jingmei Pan, Funeng Xu, Binfen Shao, Yi Wang, Xing Guo, Shaobing Zhou

**Affiliations:** ^1^ Key Laboratory of Advanced Technologies of Materials Ministry of Education School of Materials Science and Engineering Southwest Jiaotong University Chengdu 610031 China; ^2^ School of Life Science and Engineering Southwest Jiaotong University Chengdu 610031 China

**Keywords:** bacteria, bacterial components, engineered bacteria, immunotherapy, nanomaterial

## Abstract

In the past decade, bacteria‐based cancer immunotherapy has attracted much attention in the academic circle due to its unique mechanism and abundant applications in triggering the host anti‐tumor immunity. One advantage of bacteria lies in their capability in targeting tumors and preferentially colonizing the core area of the tumor. Because bacteria are abundant in pathogen‐associated molecular patterns that can effectively activate the immune cells even in the tumor immunosuppressive microenvironment, they are capable of enhancing the specific immune recognition and elimination of tumor cells. More attractively, during the rapid development of synthetic biology, using gene technology to enable bacteria to be an efficient producer of immunotherapeutic agents has led to many creative immunotherapy paradigms. The combination of bacteria and nanomaterials also displays infinite imagination in the multifunctional endowment for cancer immunotherapy. The current progress report summarizes the recent advances in bacteria‐based cancer immunotherapy with specific foci on the applications of naive bacteria‐, engineered bacteria‐, and bacterial components‐based cancer immunotherapy, and at the same time discusses future directions in this field of research based on the present developments.

## Graphical abstract

Bacteria‐based cancer immunotherapy has shown significant potential in the realm of tumor therapy, mainly because of bacterial specific targeting and preferential colonization of tumors, the abundant natural antigen library of bacteria, and the great engineerability. These high‐quality properties of bacteria have been and are being researched extensively, allowing the discovering of best immunotherapies.

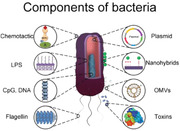

## 1 Introduction

The ongoing exploration of the tumor microenvironment (TME) in recent years has gradually unveiled a fact that the tumor immunosuppressive microenvironment is another big hurdle that needs to be overcome in cancer treatment besides those already known adverse factors that severely limit the therapeutic effect like the tumor interstitial pressure, hypoxia, nutritional deprivation, inadequate blood perfusion, immature lymphatic system, etc.^[^ ^1^ ^]^ The tumor immune microenvironment (TIME) is mainly composed of tumor cells, multiple kinds of immune cells, endothelial cells, cancer‐associated fibroblasts, cytokines and chemokines, etc., all of which constitute a complex TME that plays an important role in tumor growth, metastasis and drug resistance.^[^ ^1^, ^2^ ^]^ TIME undergoes dynamic changes along with the rapid growth of tumor, acting as a hotbed for tumor's rapid proliferation and invasion and a helper for tumor immune escape.^[^ ^3^ ^]^ In fact, it is the tumor's hypoxic environment that provides a solid foundation for this hotbed. The oxygen concentration in the tumor tissue is only 7–28 mm Hg (1–4%), while it is 40–60 mm Hg (5–8%) for the normal tissue.^[^ ^4^ ^]^ Such a hypoxic environment not only poses a huge challenge to tumor radiotherapy and chemotherapy but also directly facilitates the formation of tumor immunosuppressive microenvironment. Therefore, the extreme hypoxia of the tumor and the immunosuppressive microenvironment are two major features of malignant tumors, which can be regarded as the key for developing tumor treatments.

As an alternative idea, some researchers focused on the tumor hypoxia‐tropic nanoparticles to realize a preferential engagement in hypoxic tumor tissues.^[^ ^5^ ^]^ Although the delivery efficiency of nanoparticles can be improved in this way, its total enrichment in tumor remains at a relatively low level.^[^ ^6^ ^]^ Compared to inactive nanoparticles, bacteria have a variety of complex physiological functions (including mobility and chemotaxis, etc.), making them good therapeutic agents or vectors that have potential applications in cancer therapy.^[^ ^7^ ^]^ As early as the late nineteenth century, William Coley injected heat‐killed streptococcal organisms in conjunction of *Serratia marcescens* (“Coley's Toxins”) into tumor patients to treat their sarcomas and other malignancies, and observed tumor ablation.^[^ ^8^ ^]^ Since then, William Coley was regarded as a trailblazer in bacteria‐based cancer immunotherapy. Afterward, researchers found that certain obligate anaerobes and facultative anaerobes could preferentially colonize the hypoxic/necrotic zone inside the solid tumor after systemic administration.^[^ ^9^ ^]^ Although the mechanism behind bacteria's efficiency in targeting tumors has not yet found a consensus on the answer, it is agreed that bacteria's tumor‐targeting ability is inseparable from the hypoxia property of tumor based on the analysis of the distribution of different strains in the body. Clarifications on this issue have been provided by many researchers. Neil S. Forbes reported that more than 10 000‐fold of *Salmonella typhimurium* (*S. typhimurium*) could be accumulated in tumors compared with other organs after one week of systemic injection.^[^ ^10^ ^]^ Several mechanisms were proposed to explain the excellent specificity: a) chaotic vasculature trapped bacteria in the tumor; b) attraction to specific TME; c) preferential replication in TME. Although a pool of studies has claimed to see a great tumor‐colonization of bacteria after intravenous administration, the initial phase of bacteria escape from vessels to tumor site still remains a mystery. Sara Leschner observed a dramatic increase in tumor necrosis factor‐*α* (TNF‐*α*) in blood after the intravenous injection of *Salmonella enterica serovar typhimurium* in an ectopic transplantable tumor model, inducing the disruption of vessels in tumors.^[^ ^11^ ^]^ As a result, bacteria were flushed into the tumor with the influx of blood and were trapped inside ever since. More convincingly, authors neutralized TNF‐*α* in the serum of tumor‐bearing mice and observed retardation of blood influx together with a delay of bacterial tumor‐colonization. Therefore, TNF‐*α* and the induced hemorrhage inside the tumor play a major part at the early stage of bacterial invasion of the tumor.

**Figure 1:**
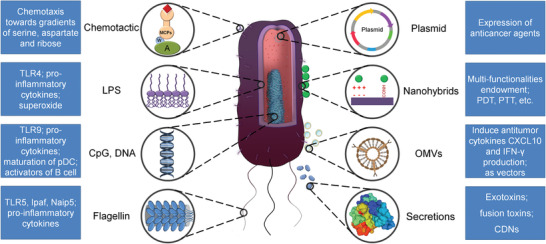
Representative features of bacteria and bacterial components, including native bacteria and engineered bacteria. Inside the blue box is the general description of every single part of bacteria and the interaction between bacterial components and the immune system.

**Figure 2:**
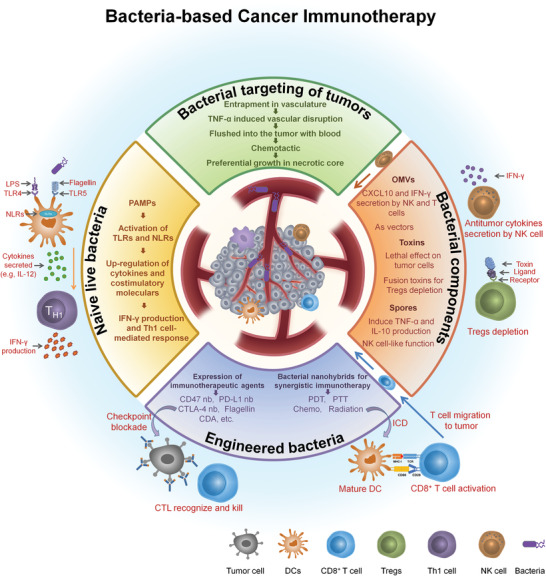
An overview of bacterial‐based immunotherapy, including the mechanism of bacteria targeting of tumors, the ways in which naive live bacteria activate the immune system, the different strategies of engineered bacteria and their connection with immunotherapy, and the activation of the immune system by different bacterial components.

Bacteria's predominant hypoxia targeting ability and specific colonization at the tumor site illustrated above, plus their immunogenicity and engineerability make them an ideal candidate for immunotherapy (**Figure** **1**).^[^ ^12^ ^]^ For example, the components of bacteria including peptidoglycan, lipopolysaccharide (LPS), lipoteichoic acid (LTA), flagellum, DNA, RNA, etc., could be identified by pattern recognition receptors (PRRs) on dendritic cells (DCs), macrophages, and neutrophils, which subsequently triggered the corresponding immune response.^[^ ^13^ ^]^ Among them, LPS, found in the outer membrane of Gram‐negative bacteria, is a typical strong immunogenic microbial‐associated molecular patterns (MAMPs), which is mainly combined with Toll‐like receptors (TLRs) on the immune cell membranes.^[^ ^14^ ^]^ The increase of LPS would cause the overexpression of interleukin‐6 (IL‐6), activation of nuclear factor kappa B (NF‐*κ*B) signaling and TLRs pathway, and phosphorylation of signal transducer and activator of transcription 3 (STAT3).^[^ ^15^ ^]^ These pathways could promote the maturation of DCs and the proliferation of immune cells, thereby enhancing anti‐tumor immunity. Similarly, bacterial flagellum would activate TLR5‐mediated innate immune response.^[^ ^16^ ^]^ For Gram‐positive bacteria without LPS and flagellum, such as *Bifidobacterium*, it could activate macrophages, natural killer (NK) cells, DCs and B lymphocytes by peptidoglycan, extracellular polysaccharides and DNA.^[^ ^17^ ^]^ Then, these effector cells could be promoted to produce immune active substances, such as IL‐1, IL‐6, IL‐12, TNF‐*α*, IFN‐*γ*, and nitric oxide (NO), and finally facilitate the anti‐tumor effect.^[^ ^18^ ^]^ Therefore, it seems that bacteria and cancer immunotherapy will inevitably be combined (**Figure** **2**). Involved with multiple immune cells, cytokines, and chemokines as stated above, bacteria‐based immunotherapy is regarded as an innovative treatment that activates and modulates the host immune system to recognize and attack cancer cells. Compared with traditional cancer treatments such as chemotherapy, radiotherapy, surgery, and phototherapy, immunotherapy could elicit the most powerful immune responses to target cancer cells with the highest accuracy and minimal side effects.

In this progress report, the applications of bacteria in cancer immunotherapy will be summarized, with specific foci on live bacteria, engineered bacteria, and bacterial components. Special attention will also be given to bacteria's delivery of immunotherapeutic agents as vectors, the mechanism behind bacteria's activation of the immune system, the synergistic treatment of bacteria, and the immune checkpoint blockade. In the end, an analysis of the hurdles that need urgent dealings and the discussion of possible future research directions will be provided.

## 2 Strategies of Bacteria‐Based Cancer Immunotherapy

### 2.1 Live Bacteria‐Based Strategy

In the late 19th century, the anti‐tumor effect of Coley's toxins made people realize for the first time the huge potential of bacteria in tumor treatment.^[^ ^8^, ^19^ ^]^ Although it was temporarily shelved by the subsequent surge of radiotherapy, there have been a wide range of emerging studies on its potential anti‐tumor application in recent decades. Bacteria's advantages in cancer treatment lie in partly their role as vectors in delivering therapeutic agents to the tumor site and partly their capabilities of interacting with the immune system and stimulating it to recognize and destroy tumor cells.^[^ ^20^ ^]^ In principle, bacteria can be introduced into the host through intravenous, subcutaneous, and intratumoral injection.^[^ ^21^ ^]^ These bacteria disperse in various parts of the host after injection, including solid tumors, and normal organs.^[^ ^22^ ^]^ However, the number of bacteria originally distributed in the vasculature and normal tissues would drop sharply within a few hours or days due to the rich oxygen in the physiological environment and the elimination of the immune system, and eventually would be completely eliminated to avoid the potential toxicity to the host.^[^ ^23^ ^]^ Bacteria that initially reach a solid tumor can move to the hypoxic necrosis core area of the tumor through various mechanisms (such as chemotaxis).^[^ ^24^ ^]^ The hypoxic microenvironment in tumor and the nutrients released from the dead tumor cells facilitate the massive proliferation of anaerobic bacteria. Moreover, the immunosuppressive TME prevents the immune system from clearing the bacteria in the tumor at the early stage. Then, these multiplied bacteria will activate the host's immune system, causing a large amount of immune cells to infiltrate within tumors.^[^ ^25^ ^]^

It is worth noting that the underlying mechanisms in the immune system for bacteria and host to interact with each other depend on bacterial strains and tumor models. For example, Listeria infection can directly kill tumor cells through the activation of nicotinamide adenine dinucleotide phosphate (NADP^+^) oxidase and elevated intracellular Ca^2+^ amount.^[^ ^26^ ^]^ Both of the two mechanisms can form reactive oxygen species (ROS), which is a highly cytotoxic free radical.^[^ ^27^ ^]^ ROS can initiate the immunogenic death of tumor cells, then activate CD8^+^ T cells to eliminate residual tumor cells, and finally prevent metastases. Meanwhile, *Listeria* can infect bone marrow‐derived suppressor cells (MDSCs) at the tumor site, causing a significant quantity decrease in MDSC and subsequently transforming the immunosuppressive microenvironment to an immunostimulatory status. Moreover, IL‐12 can be produced upon the transformation of the remaining infected MDSCs into immunostimulatory phenotype, which can improve the T cells’ and NK cells’ response.^[^ ^28^ ^]^

*Clostridium Bacillus* destroys tumor cells mainly by secreting some exotoxins. For instance, hemolysins and phospholipases can kill tumor cells by destroying their membrane structure.^[^ ^29^ ^]^ *Clostridium* can activate tumor apoptosis by triggering the release of tumor necrosis factor‐related apoptosis‐inducing ligand (TRAIL) from polymorphonuclear neutrophils. Studies have reported that matrix metalloproteinase 8 (MMP‐8) played a vital role in this release process.^[^ ^30^ ^]^ Moreover, the early expansion of *Clostridium* in solid tumors could lead to intratumoral infiltration of granulocytes and macrophages, and the increased secretion of chemokines would further trigger the adaptive immunity and recruit immune cells (e.g., CD8^+^ T cells) to the tumor site.^[^ ^25a^ ^]^

**Figure 3:**
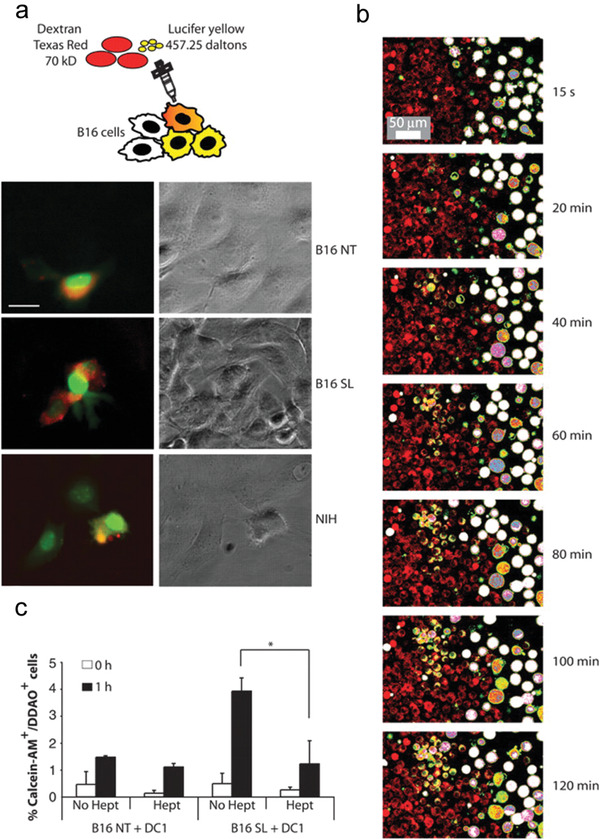
Bacteria‐induced Cx43 up‐regulation interrelated with establishment of gap junctions. a) B16 NT: not infected B16 cells; B16 SL: infected B16 cells with *Salmonella*; NIH: Untreated NIH 3T3 cells. GJ‐diffusible dye Lucifer yellow (green) and the nontransferable dye dextran–Texas Red (red, mark microinjected cells). Scale bar, 20 µm. b) Confocal microscopy images. B16 cells were infected with *Salmonella* and stained with calcein‐AM (green/white), whereas DCs were stained with DDAO (red). *Salmonella*‐infected B16 cells and DCs were coincubated for 1 h before continuously observation on confocal microscopy. c) Only *Salmonella*‐infected B16 cells could interact with DCs through a GJ‐dependent mechanism.Reproduced with permission.^[^ ^36a^ ^]^ Copyright 2010, The American Association for the Advancement of Science.

*Salmonella* is another type of bacteria that has been widely studied in the field of cancer therapy; its interaction with the immune system has been clarified as well.^[^ ^31^ ^]^ After infecting tumor cells, *Salmonella* directly destroys tumor cells either by initiating autophagy pathway or inducing apoptosis.^[^ ^32^ ^]^ Furthermore, pathogen‐associated molecular patterns (PAMPs) such as flagellin and LPS released from *Salmonella* can be captured and recognized by antigen‐presenting cells (APCs).^[^ ^12^, ^33^ ^]^ Flagellin can promote the maturation of APCs and up‐regulate pro‐inflammatory cytokines (e.g., IL‐12) and co‐stimulatory molecules (e.g., CD40) by binding and activating TLR5 and Nod‐like receptors (NLRs) on APCs.^[^ ^34^ ^]^ These inflammatory mediators can subsequently stimulate the secretion of interferon‐gamma (IFN‐*γ*) and the T helper type 1 (Th1) cell‐mediated immune response. In this process, macrophages and DCs secrete pro‐inflammatory IL‐1*β* and TNF‐*α* under the stimulation of LPS‐induced TLR4 signaling and tumor cell debris.^[^ ^25b^ ^]^ Flagellin has been found to be capable of activating NK cells to produce IFN‐*γ* through a TLR‐independent pathway involving IL‐18 and Myeloid differentiation factor 88 (MyD88), and the produced IFN‐*γ* will lower the frequency of CD4^+^ CD25^+^ regulatory T cells (Tregs) in the TME.^[^ ^16^, ^35^ ^]^ Studies have shown that *Salmonella* infection can lead to the up‐regulation of connexin 43 (Cx43) in human and murine melanoma cells (**Figure** **3**). Thus, the functional gap junctions between melanoma cells and adjacent DCs are formed.^[^ ^36^ ^]^ Tumor cells can deliver antigenic peptides to DCs through gap junctions. DCs present these peptides on the cell surface to activate cytotoxic T cells against tumor antigens and suppress tumor proliferation.^[^ ^36^, ^37^ ^]^ The Cx43‐dependent cross‐presentation pathway induced by *Salmonella* infection is more effective than the traditional way that DCs pick up and process antigens themselves. In a phase I human clinical trial, an attenuated *S. Typhimurium* strain (VNP20009) was tested on 24 patients with metastatic melanoma and one patient with metastatic renal carcinoma.^[^ ^38^ ^]^ Patients received 10^6^ to 10^9^ cfu m^−^² of VNP20009 through systematic administration and then were evaluated for dose‐related toxicities, selective replication within tumors, and anti‐tumor effects. The maximum‐tolerated dose was determined as 3.0  ×  10^8^ cfu m^−^². And some tumor colonization was observed at the highest tolerated dose. Although dose‐related increase of several proinflammatory cytokines (such as IL‐1*β*, TNF‐*α*, IL‐6, and IL‐12) was observed, no patients experienced objective tumor regression. The clinical results suggest that additional methods were required to enhance the efficacy and reduce the toxicity.

Although native anaerobic and facultative anaerobic bacteria are excellent at colonization in tumor and killing tumor cells through immune cells’ infiltration, the single functionality and ineffective therapeutic action seriously limit their development and application. Besides, some toxic and side effects have been observed after systemic injection of native bacteria.^[^ ^39^ ^]^ With the advancement of synthetic biology, bacteria have been engineered for attenuation to ensure safety.^[^ ^40^ ^]^ After decades of rapid development, this transformative approach not only enables researchers to focus on the attenuation but also adopts a variety of engineering methods to develop intelligent bacteria for a wide range of applications.^[^ ^41^ ^]^

### 2.2 Engineered Bacteria‐Based Strategy

#### 2.2.1 Engineered to Produce and Express Immunotherapeutic Agents

Immunotherapeutic agents include monoclonal antibodies, chemokines, and cytokines, etc. Due to their large molecular weight and good water solubility, it is particularly difficult even for nano carriers to deal with the targeted delivery of these proteins. The rapid development of synthetic biology enables researchers to creatively develop engineered bacteria that produce and express multiple immunotherapeutics. These bacteria can efficiently and safely express immunotherapeutic agents in the local tumor, thereby triggering a strong anti‐tumor immune response. This kind of immune stimulation usually results from the joint action of two aspects. One is that the expressed immunotherapeutic agent specifically targets a certain part of the immune system, while the other is that the bacteria supplement the immune stimulation through PRRs, thereby generating maximum anti‐tumor immunity. Another benefit of this method is to reduce the off‐target of the therapeutic agent, hence reducing the toxic side effects.

##### Nanobody Antagonist

**Figure 4:**
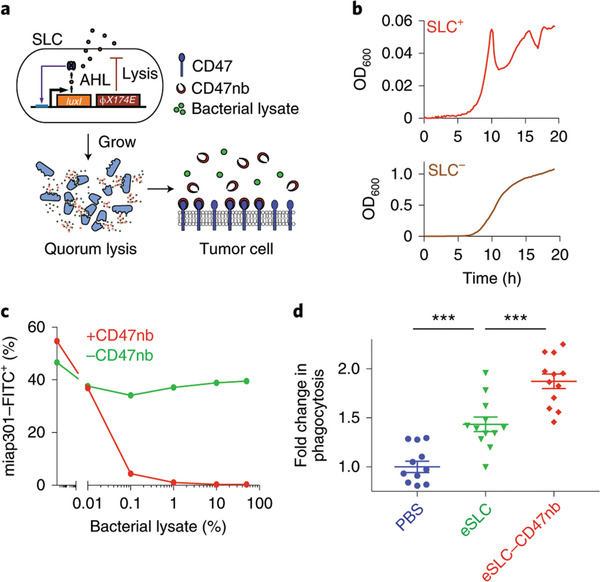
Quorum‐induced release of anti‐CD47 blocking nanobodies by SLC‐encoded bacteria. a) *E. coli* with SLC reach a quorum and activate the phage‐lysis protein *ϕ*X174E, resulting in bacterial lysis and release of anti‐CD47 blocking nanobodies. b) Growth dynamics of SLC^+^ and SLC bacteria over 20 h. Reproduced with permission.^[^ ^42^ ^]^ Copyright 2019, Springer Nature.

Recently, Sreyan Chowdhury et al. reported an engineered *Escherichia coli* (*E. coli*) strain to release nanobody antagonist of CD47 (CD47nb), which is an anti‐phagocytic receptor highly expressed in some human cancer types.^[^ ^42^ ^]^ The *E. coli* strain used in this work comprised a synchronized lysis circuit (SLC) in which lyse bacteria released its therapeutic products once reaching a critical density (**Figure** **4**). This method takes advantage of the "quorum sensing" effect of bacteria in nature and improves the previously developed bacterial therapy.^[^ ^43^ ^]^ In the original method, bacteria could autonomously produce drugs, which means that they might produce and release therapeutic substances in non‐designated areas of the body. In contrast, the bacteria in the new method would destruct themselves and release the payload only in the tumor site as they just reached the critical density there, preventing the uncontrolled growth of bacteria in other unexpected tissues. Owing to potent nanobody‐mediated blockade of CD47, authors observed increased activation of tumor‐infiltrating T cells, durable and systemic anti‐tumor immunity, and rapid tumor regression.

**Figure 5:**
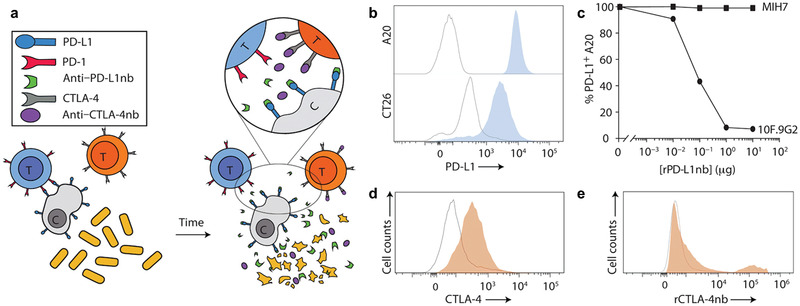
Engineering design and characterization of a bacteria‐based cancer treatment platform for in situ release of immune checkpoint inhibitor. a) Mechanism illustration by which engineered bacteria controllably and sustainably release PD‐L1 and CTLA‐4 nanobodies. b) Flow cytometric data of PD‐L1 expression on A20 and CT26 cells (outlined peaks, negative control; blue‐filled peaks, PD‐L1). c) Binding behavior of rPD‐L1nb to the 10F.9G2 and MIH7 PD‐L1 epitopes on A20 cells. d,e) Isolated splenocytes from naive C57BL/6 mice were analyzed by flow cytometry for d) intracellular CTLA‐4 expression (outlined peak, unstimulated CD3^+^ splenocytes; orange‐filled peak, PMA/ionomycin‐simulated CD3^+^ splenocytes) and e) rCTLA‐4nb binding to extracellular CTLA‐4 (outlined peak, secondary anti‐HIS antibody alone gated on CD3^+^ splenocytes; orange‐filled peak, rCTLA‐4nb gated on CD3^+^ splenocytes). Reproduced with permission.^[^ ^46^ ^]^ Copyright 2020, The American Association for the Advancement of Science.

Immune checkpoint blockade as the most typical clinical immunotherapy method, has displayed a series of achievements.^[^ ^44^ ^]^ However, the high probability of immune‐related side effects such as fatigue, skin rashes, endocrine disorders, and hepatic toxicities limits its wider application.^[^ ^45^ ^]^ To solve this problem, the key is to efficiently deliver immune checkpoint inhibitors to the tumor site and ensure an enduringly constant release of them. As mentioned above, bacteria can specifically colonize tumors and selectively grow in the necrotic tumor core. These characteristics of bacteria and their good programmability make them ideal carriers to deliver immune checkpoint inhibitors to tumor. Candice R. Gurbatri and colleagues engineered *E. coli Nissle 1917* (*EcN*) via transforming the high‐copy plasmid carrying programmed death‐ligand 1 (PD‐L1) nanobody and cytotoxic T‐lymphocyte‐associated protein 4 (CTLA‐4) nanobody sequences to locally and controllably express PD‐L1 and CTLA‐4 antagonists (**Figure** **5**).^[^ ^46^ ^]^ To ensure the maximum therapeutic efficacy, the authors employed SLC to release antagonists as well. In multiple syngeneic mouse models, partial or complete regression of tumors was observed with intratumoral injections of engineered *EcN*. According to immunophenotype study, the number of both intratumoral activated CD8^+^ T cells and conventional CD4^+^ T cells was increased when the number of Tregs was decreased, suggesting a shift from immunosuppressive TIME to a responsive state and triggering a robust adaptive immune response with this intelligent engineered system.

##### PAMPs

Flagellin, as a component of some certain bacteria, can be used as an immunostimulator for tumor immunotherapy. Flagellin is a globular protein that constitutes the flagellum fiber of bacteria and is mainly composed of three parts: matrix bodies, hooks and filaments.^[^ ^47^ ^]^ The matrix body rivets on the bacterial membrane and acts like a reversible rotary motor. The filament is connected to the matrix body through a hook, and its function is equivalent to a propeller. It is arranged in a hollow cylinder, forming filaments in the flagella of bacteria. Flagellin presents itself in almost all flagellar bacteria with a molecular weight of about 30 000–60 000 Daltons. Flagellin has four domains: D0, D1, D2, and D3, which are arranged in order from inside to outside. Among them, D0 and D1 domains are *α*‐helix structures, while the structures of D2 and D3 domain are mainly *β*‐sheets and *β*‐curls.^[^ ^48^ ^]^ The main physiological function of flagellin in bacteria is to form flagella – the organs that enable bacteria to move freely under the liquid environment.^[^ ^49^ ^]^

**Figure 6:**
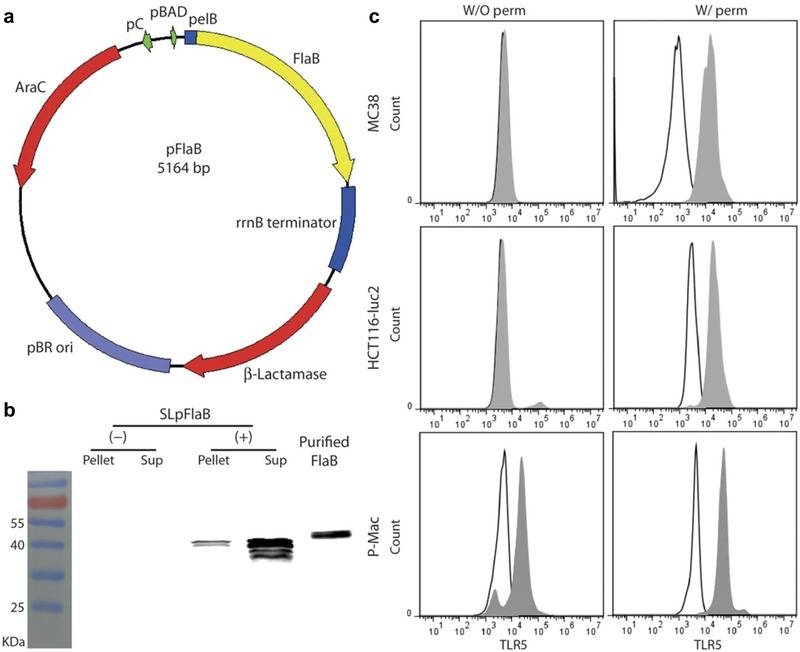
Engineering strategy of FlaB‐expressing bacteria and analysis of TLR5 expression on cancer cells. a) Schematic map of engineered plasmid pFlaB containing 5164 base pair. b) In vitro analysis of bacterial expression of FlaB by immunoblot. c) Flow cytometry data of TLR5 expression in MC38, HCT116‐luc2, and peritoneal macrophages with permeabilized (W/ perm) and nonpermeabilized (W/O perm) conditions. Outlined peaks: isotype control; gray‐filled peaks: stained by TLR5 antibody. Reproduced with permission.^[^ ^57^ ^]^ Copyright 2017, The American Association for the Advancement of Science.

The receptors of human immune cells that can recognize flagellin include membrane receptor TLR5 and intracellular receptor interleukin converting enzyme activator Ipaf and Nod‐like receptor apoptosis‐inhibitory protein‐5 (Naip5).^[^ ^50^ ^]^ As one of the 11 TLRs, the TLR5 receptor is present on the membrane of epithelial cells, monocytes, DCs, and T cells; Ipaf and Naip5 belong to the NLRs family and are mainly present in the cytoplasm of monocytes.^[^ ^50a^ ^]^ TLR5 can recognize the conserved D0 domain of flagellin and then recruit the downstream signals (MAP enzymes including p38 and IkB) by activating MyD88 after dimerization.^[^ ^51^ ^]^ In the last step, it activates NF‐*κ*B and regulates cells from cellular transcription. Therefore, flagellin can also be used as an immune adjuvant, mainly based on the ability to activate DCs and non‐intrinsic immune cells (such as epithelial cells and lymphatic stromal cells).^[^ ^52^ ^]^ The interaction of flagellin and TLR5 results in the expression of varied proinflammatory cytokines, NO, H_2_O_2_, chemokines, and host defense proteins.^[^ ^53^ ^]^ It is revealed that engineered *Salmonella* in the TME exerts a strong anti‐tumor efficacy through the activation of the TLR4 and TLR5 pathways. The engineered flagellin‐secreting *Salmonella* has a greater inhibitory effect on tumors than free *Salmonella*, indicating that TLR4 and TLR5 pathways have a synergistic effect on tumor growth inhibition.^[^ ^54^ ^]^ Moreover flagellin is also closely related to tumor immunosuppressive microenvironment. The inhibitory signals produced from tumor cells in the TME effectively prevent the surveillance of the immune system. MDSCs can recruit regulatory T cells and inhibit the activation of NK cells and effector T cells.^[^ ^55^ ^]^ Intratumoral injection of flagellin can effectively reduce the number of MDSCs in the tumor. In TME, tumor‐associated M2‐like macrophages promote tumor growth by inhibiting DCs maturation, down‐regulating the expression of major histocompatibility complex (MHC) molecules, and recruiting regulatory T cells.^[^ ^56^ ^]^ Interestingly, it has been proved that flagellin can reverse the tumor immunosuppressive microenvironment into immune‐responsive TIME by the polarization of M2‐like macrophages to M1‐type macrophages. Jin Hai Zheng et al. reported that an engineered attenuated *S. typhimurium* strain could successfully suppress the tumor growth and metastasis in murine colon and melanoma models through a secretion of Vibrio vulnificus flagellin B (FlaB) (**Figure** **6**).^[^ ^57^ ^]^ The underlying mechanism can be interpreted in the following manner: TLR4 signaling is a key to suppress tumor growth by inducing the activation and infiltration of immune cells mediated by FlaB‐secreting bacteria, whereas TLR5 signaling is capable of augmenting the host reactions. The results provided evidence for the fact that locally produced FlaB by engineered bacteria could induce potent anti‐tumor immunity and prolong the survivability of tumor‐bearing mice.

##### STING Agonists

**Figure 7:**
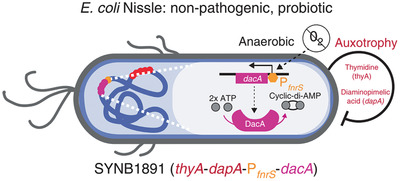
Schematic diagram of engineering strategy for finalized SYNB1891 bacteria strain. Reproduced with permission.^[^ ^61^ ^]^ Copyright 2020, Springer Nature.

Like TLRs, as an important part of the innate immune system, the stimulator of interferon genes (STING) pathway has received elevated attention since its discovery.^[^ ^58^ ^]^ STING can be activated by binding to cyclic dinucleotides (CDNs) and initiating a potent pro‐inflammatory cytokines production, including type I IFNs. Numerous studies have shown that STING agonists exhibit potent anti‐tumor activity, opening the way for developing STING‐based cancer immunotherapy.^[^ ^59^ ^]^ However, although the STING agonists can activate the STING pathway to trigger an anti‐tumor immune response in cancer treatment, it still has some shortcomings, such as off‐target effects and toxicity.^[^ ^60^ ^]^ Therefore, the use of synthetic biology technology to engineer bacteria to produce and release STING agonists after colonization on tumor sites has become a preferred solution. Also, as a good source of immune stimulation through PRRs, bacteria can activate complementary immune pathways to provide better curative effects. In a recent report, Daniel S. Leventhal and co‐workers engineered a strain of *E. coli Nissle* (referred to as *SYNB1891*) to express STING‐agonist cyclic diAMP (CDA) (**Figure** **7**).^[^ ^61^ ^]^ After intratumoral injection of SYNB1891 in murine tumor, a high‐level expression of CDA in tumor tissue was observed. Besides, type I IFN and pro‐inflammatory cytokines were up‐regulated in a dose‐dependent manner, including TNF‐*α*, IL‐6, IL‐1*β*, IFN‐*γ*, and granulocyte‐macrophage colony‐stimulating factor (GM‐CSF). These results confirmed the great potential of *SYNB1891* in triggering an anti‐tumor immune response. Moreover, two distinct murine tumor models (B16F10 and A20) and two different genetic backgrounds mice (C57BL/6 and BALB/c) were carried out and verified that *SYNB1891* could trigger effective anti‐tumor immunity and immunological memory.

##### Cytokines

Cytokines are widely studied due to their intrinsic functions to promote the activation and proliferation of immune cells to achieve anti‐tumor effects. However, systemic administration of cytokines causes serious side effects, limiting its clinical application. Among different routes to deliver cytokines to the tumor site, engineered tumor‐targeting bacteria to express cytokines have multiple advantages such as high‐level specificity but less side effects. Markus Loeffler and co‐workers engineered attenuated *S. typhimurium* to express LIGHT, which is a TNF‐family cytokine that has been demonstrated to generate promising anti‐tumor activity.^[^ ^62^ ^]^ In several mouse tumor models, authors observed satisfactory tumor suppression with no significant toxicity. And the TME analysis demonstrated that the efficacy was associated with the massive inflammatory cell infiltration into tumors including the elevated frequency of DCs. Based on this novel strategy, authors further armed attenuated *S. typhimurium* with the ability to synthesize IL‐18, which is an IFN‐*γ*‐inducing factor with multiple functions related to the proliferation and activation of immune cells.^[^ ^63^ ^]^ In preclinical mouse cancer models, the endowment of IL‐18‐producing ability greatly increased *S. typhimurium*’s anti‐tumor activity. The mechanism was associated with increased frequency of tumor infiltrating CD3^+^/CD4^+^ T cells and DX5^+^ NK cells. Meanwhile, several immunostimulatory and inflammatory cytokines were brought into play in tumor suppression, including GM‐CSF, IL‐1*β*, and TNF*α*. These preclinical findings show that cytokine‐producing bacteria could elicit strong immune response with no side effects, suggesting a novel strategy for bacteria‐based cancer immunotherapy.

#### 2.2.2 Engineered for Other Combination Therapy

In addition to the use of genetic technology to engineer bacteria, the integration of bacteria and nanomaterials for multifunctional synergistic therapy is considered to be a promising treatment.^[^ ^40c^ ^]^ Nanomaterials can achieve a variety of functions in cancer therapy due to different synthesis methods and encapsulated drugs.^[^ ^64^ ^]^ Therefore, compared with the genetic modification of bacteria to secrete therapeutic agents, it seems more direct to integrate nanomaterials with other diverse functions in the outer membrane of bacteria. The biggest benefit brought by this is the easy preparation and the low cost; however, there still exist some shortcomings.

First of all, bacteria can target tumors partly because the outer membrane of bacteria is rich in outer membrane proteins, some of which are used to sense the surrounding environment and perform chemotactic movement of certain specific molecular signals. When integrating nanomaterials into the outer membrane of bacteria, the chemotactic function of the bacteria will be inevitably affected if a dense and continuous shell is formed to completely enclose the bacteria, thereby weakening the targeting ability of bacteria. Another consideration lies in the strength of the connection between nanomaterials and bacterial membranes. The engineered bacteria need to face a complex physiological environment, such as high‐speed blood flow, high salinity, a variety of metal ions, and abundant fibrin.^[^ ^65^ ^]^ To prevent the early detachment of nanomaterials in the body fluid environment and the loss of the expected versatility on the collaborative treatment platform, a strong connection between the nanomaterials and bacteria is particularly important. Therefore, researchers mostly use stable amide bonds to connect the two bodies to prevent functional materials from falling off in advance. Furthermore, the proper function should be selected to integrate with the ontology of bacteria to produce a synergy rather than antagonism. The response to external stimuli allows the integrator to act on its orders and achieve a controlled treatment. Light irradiation, magnetic field, and ultrasonic are the commonly used externally applied stimuli. Among them, light irradiation is the most‐widely used and mature stimulus in tumor treatment.

**Figure 8:**
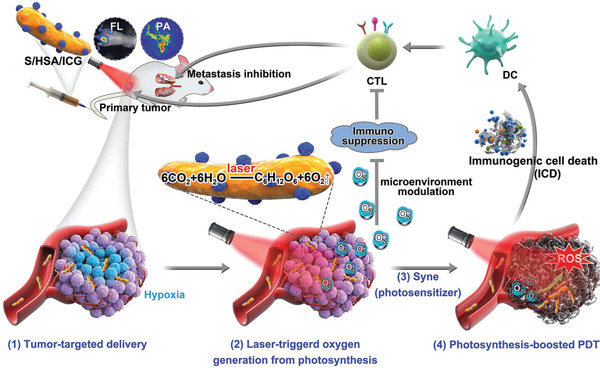
Schematic depiction of nanophotosensitizer conjugated *Syne *(S/HSA/ICG) as an in situ photocatalyzed oxygen generation platform for metastatic cancer immunogenic PDT. Synthesized nanophotosensitizer HSA/ICG was conjugated to *Syne *through amide bonds. Due to the tumor‐targeted effect of *Syne *and HSA, *Syne *and ICG were greatly accumulated inside the tumor. With laser radiation, high oxygenic level was created by *Syne*‐induced photosynthesis and further facilitated the ROS production in PDT. Meanwhile, the production of oxygen relieved tumor hypoxia and reversed tumor immunosuppressive microenvironment, thus enhanced PDT efficacy and stimulated the ICD‐mediated antitumor immunity. Reproduced with permission.^[^ ^69^ ^]^ Copyright 2020, Wiley‐VCH.

Phototherapy, including photodynamic therapy (PDT) and photothermal therapy (PTT), has gained increasing attention due to its precision and non‐invasion.^[^ ^66^ ^]^ Applying engineering methods to integrate phototherapy nanoparticles with bacteria can effectively achieve precise tumor targeting and powerful primary tumor ablation. Moreover, the sequent anti‐tumor immunity could achieve multi‐functional combination therapy to maximize the therapeutic effect of tumors. However, although the tumor's hypoxia microenvironment provides an excellent condition for bacterial intrinsic tumor targeting ability, it greatly limits the therapeutic effect of PDT.^[^ ^67^ ^]^ The photosynthetic bacteria were previously applied for cardiovascular disease because of its highly efficient oxygen generation.^[^ ^68^ ^]^ The light‐controllable growth and photosynthesis of bacteria could relieve tumor hypoxia for enhanced immunogenic PDT. Lanlan Liu et al. engineered photosynthetic bacteria (*Synechococcus 7942*, *Syne*) by integrating photosensitizer‐encapsulated nanoparticles on the bacterial surface via amide bonds (**Figure** **8**).^[^ ^69^ ^]^ The constructed biomimetic system enabled the improved accumulation of photosensitizers to rely on bacterial intrinsic targeting ability. When irradiated by 660 nm laser, *Syne* could generate oxygen continuously via photosynthesis and thus greatly ameliorate the tumor hypoxia, leading to more ROS production. The photosynthesis‐boosted PDT can not only suppress the primary tumor growth but also eliminate metastatic tumors and prevent tumor recurrence by reversing the immunosuppressive TME to an immune‐responsive state even against triple‐negative breast cancer mouse model. Different from directly delivering immunotherapeutic agents to tumor tissue, PDT could evoke anti‐tumor immunity by inducing immunogenic cell death (ICD) that involved the up‐regulated expression of calreticulin (CRT) on the cell surface.^[^ ^70^ ^]^ The CRT exposure on the membrane of tumor cells can promote the activation and maturation of DCs. Interestingly, not only CRT but also ROS produced by PDT could accelerate the maturation of DCs.^[^ ^71^ ^]^ In addition to producing oxygen locally, *Syne* as immunogenic bacteria can trigger moderate immune activation through upregulating the percentage of MHC II and increasing the production of IL‐12p40. Meanwhile, improved tumor hypoxia promotes the infiltration of effector T cells to the tumor since T cells tend to avoid hypoxic areas and head to neighboring normoxic regions in TME.^[^ ^72^ ^]^ Photosynthesis‐boosted immunogenic PDT shows a great ability to ameliorate the TIME by elevating the percentage of immune effector cells such as CD8^+^ T cells, CD4^+^ T cells, and NK cells, and decreasing the number of immunosuppressive cells such as Tregs, MDSCs, and M2‐like tumor‐associated macrophages (TAM) which are important for suppressing anti‐tumor immunity.^[^ ^73^ ^]^

**Figure 9:**
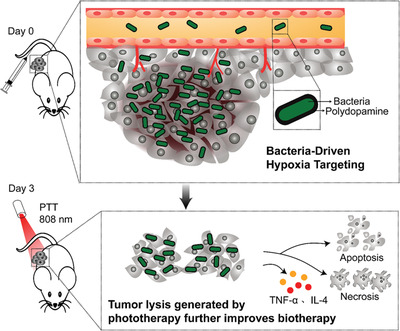
Bacteria‐driven hypoxia targeting and PTT‐induced tumor lysis generation improve biotherapy. Reproduced with permission.^[^ ^75^ ^]^ Copyright 2018, American Chemical Society.

**Figure 10:**
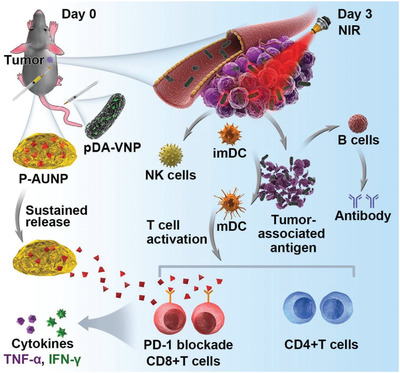
The triple‐combination of bacteria, PTT, and sustained release of PD‐1 blockage therapy initiated potent antitumor immunity in vivo. Reproduced with permission.^[^ ^76^ ^]^ Copyright 2019, Wiley‐VCH.

Some researchers also tried to engineer the bacteria with PTT photosensitizer. PTT, as a non‐invasive cancer treatment, can cause strong tumor ablation and simultaneously induce heat shock proteins (HSP) produced by tumor cells, which is a family of proteins with moderate immunostimulant function.^[^ ^74^ ^]^ Due to the good biocompatibility and biodegradability, photosensitizer polydopamine (pDA) is a promising candidate for PTT. To achieve the combination of hypoxic tumor targeting of bacteria and photothermal behavior of pDA, researchers have integrated pDA into the surface of anaerobe *Salmonella* strain *VNP20009* to make it a thicker artificial outer membrane (**Figure** **9**).^[^ ^75^ ^]^ The engineered bacteria with pDA are still active, and its tumor targeting ability has not diminished. The combination of biotherapy and PTT achieves highly effective tumor suppression and enhances the therapeutic effect by promoting the production of TNF‐*α* and IL‐4 to trigger cellular immunity and humoral immune response. In a later report, the authors combined the immune checkpoint blockade with photothermal functional bacteria to achieve innovative triple therapy (**Figure** **10**).^[^ ^76^ ^]^ In this study, authors chose peptide AUNP‐12 rather than antibodies (e.g., pembrolizumab and nivolumab) to block programmed cell death protein 1 (PD‐1). AUNP‐12 has been proven to perform well on the antagonism of PD‐1 by subcutaneous administration and showed a safe toxicological profile.^[^ ^77^ ^]^ Although peptide antagonists have better tumor penetration properties, short‐term retention in tumor tissues limits their application. Therefore, the authors applied subcutaneous injection of AUNP‐12‐loaded phospholipid‐based phase separation gel (PPSG) near the tumor to achieve long‐term sustained release of the antagonist. With gel sustained‐release system, the immune checkpoint inhibitor could stay in the tumor tissue for up to 42 days, enhancing the anti‐tumor effect of triple combination therapy. Compared with the *VNP20009* treatment alone, the amount of TNF‐*α* at the tumor in the triple therapy group was 7.9× that of the bacterial treatment group. Moreover, every element in the triple therapy contributed to the immune activation of the tumor. With the release of tumor‐associated antigens, the maturation of DCs, the production of proinflammatory cytokines, the recruitment and functioning of T cells, and the promotion of NK cells, the triple therapy including pDA engineered bacteria and immune checkpoint blockade achieved full‐stage promotion of anti‐tumor immunity and exhibited effective suppression of advanced melanoma.

In addition to genetic engineering, the hybridization of bacteria and nanomaterials is another strategy to functionalize bacteria. The appropriate modification of nanomaterials on the surface of live bacteria would not affect the intrinsic tumor‐targeting ability of bacteria. The additional functions (e.g., PDT, PTT etc.) achieved by the introduction of nanomaterials have a synergistic effect on bacterial therapy and enhance the therapeutic effect. Usually, such combination therapy can simultaneously enhance several aspects of anti‐tumor immune activation, leading to the reversal of the tumor immunosuppressive microenvironment and maximizing anti‐tumor immunity. Although the combination of phototherapy with bacteria‐based cancer immunotherapy has made great achievements, the intrinsic drawbacks of phototherapy including the insufficient light penetration depth and the potential phototoxicity to the skin need to be considered in future studies.

### 2.3 Bacterial Component and Product‐Based Strategy

#### 2.3.1 Bacterial Outer Membrane Vesicles

**Figure 11:**
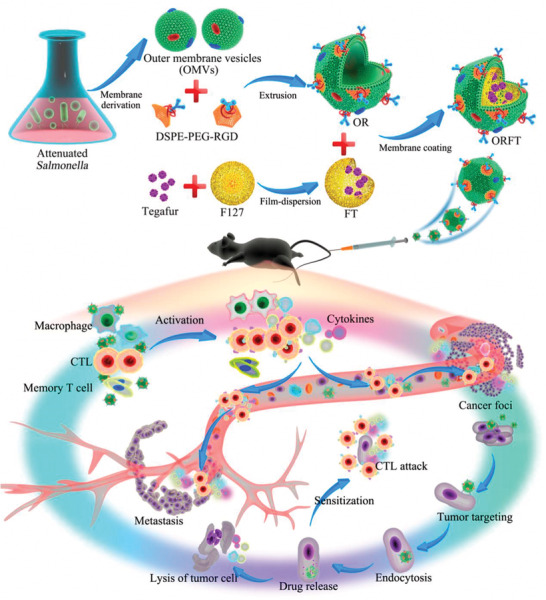
Schematic illustration of the bioengineering process of multi‐functional OMV‐coated polymeric micelles and in vivo mechanisms of immunotherapy for antitumor immunity and metastasis prevention. Reproduced with permission.^[^ ^87^ ^]^ Copyright 2020, American Chemical Society.

**Figure 12:**
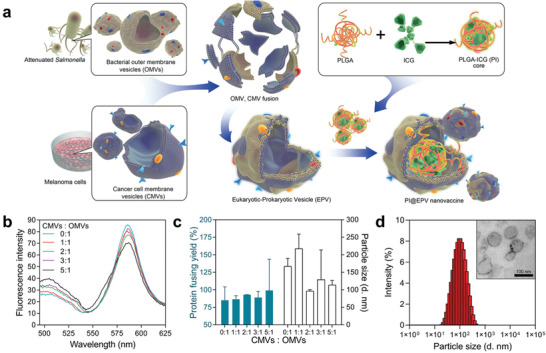
Fabrication and characterization of PI@EPV nanovaccine. a) Schematic illustration of fabrication steps of eukaryotic‐prokaryotic vesicles coated PI@EPV nanovaccine. b) A pair of Förster resonance energy transfer (FRET) fluorescence dyes DiL and DiO is selected to evaluate the fusion process of two types of membranes. c) Quantification of protein loading yields and hydrodynamic sizes of EPVs in PBS with a set of weight ratios of OMVs versus CMVs. d) Hydrodynamic size and morphological characterization of EPV (CMVs: OMVs = 2:1). Reproduced with permission.^[^ ^88^ ^]^ Copyright 2020, Wiley‐VCH.

Outer‐membrane vesicles (OMVs) are nano‐sized spherical vesicles produced by Gram‐negative bacteria.^[^ ^78^ ^]^ OMVs mainly consist of cellular components of the bacterial periplasm and the outer membrane, including membrane lipids, proteins, LPS, peptidoglycans (PG), and virulence factors.^[^ ^79^ ^]^ Some intracellular components may also be wrapped, such as some intracellular proteins, DNA, RNA, ions, metabolites, signaling molecules, and enzymes.^[^ ^80^ ^]^ The mechanism of OMVs production remains unclear. There are currently three hypotheses widely recognized: 1) the accumulation of phospholipid in the outer leaflet of the bacterial outer membrane and regulation by VacJ/Yrb ATP‐binding cassette transport system for most Gram‐negative bacteria result in the OMVs formation;^[^ ^81^ ^]^ 2) the cross‐linking of the outer membrane and the PG layer. The cell wall of Gram‐negative bacteria is composed of outer membrane and PG, while lipoprotein crosslinks them with covalent bonds to maintain the integrity of the envelope structure. When decomposition or abnormal synthesis of the PG layer happens, a small part of the outer membrane would dissociate from the PG layer and protrude out of the cell to form OMVs;^[^ ^82^ ^]^ 3) the accumulation of the misfolded proteins and abnormal envelope components in the periplasm would promote the formation of OMVs. These unexpected substances may reduce the integrity of the envelope, thereby separating the PG layer and the outer membrane layer. As a result, the buds are extruded outward to form OMVs.^[^ ^83^ ^]^ OMVs containing a large number of MAMPs (such as LPS, outer membrane proteins, PG, flagellin, RNA, DNA, etc.) can interact with host PRRs to induce innate immune responses. Owing to the abundant natural adjuvant components in OMVs, systemic injection of OMVs or siRNA‐packaged OMVs from a mutant *E. coli* strain has been reported to up‐regulate the production ofTNF‐*α*, IL‐6, IFN‐*γ*, and anti‐tumor cytokines CXCL10, which are closely related to the anti‐tumor immunity.^[^ ^84^ ^]^ Among a variety of MAMPs, the LPS stands out like a star. It is composed of lipid A, core polysaccharide and O‐antigen. O‐antigen is a kind of polysaccharide exposed on the surface of the bacterial outer membrane, which is an epitope of bacterial cell antigens.^[^ ^85^ ^]^ Lipid A is regarded as the active biological center of LPS; it can cause strong inflammation and regulate immune response such as stimulating immune cells to produce antibodies against multiple antigens. However, current studies have shown that excess LPS would cause immunosuppressive reactions, and blocking lipid A can remove endotoxin activity and reduce immunosuppression.^[^ ^86^ ^]^ OMVs as immune adjuvants need to appropriately reduce the toxicity of LPS, that is, knocking out the activity of lipid A through genetic modification to achieve the purpose of attenuation. Oh Youn Kim et al. knocked out the msbB gene encoding endotoxin in *E. coli* to avoid the immunosuppression caused by lipid A, and found that the use of G‐bacteria OMVs in mouse colon cancer model could inhibit tumor growth.^[^ ^84b^ ^]^ The outer membrane protein of vesicles stimulated NK cells and T cells to produce INF‐*γ*, which in turn has growth inhibitory effect on tumor. In some cases, naturally‐produced OMVs were used directly as carriers for drug delivery. For example, immunomodulatory molecules, photosensitizers, and chemo drugs can be loaded in OMVs and then transported to tumor, achieving a combination of immunotherapy and chemotherapy/phototherapy. Qi Chen et al. coated DSPE‐PEG‐RGD‐ hybridized bacterial OMVs on drug‐loaded polymer micelles to produce an innovative nanomedicine for effective cancer immunotherapy and metastasis prevention (**Figure** **11**).^[^ ^87^ ^]^ The authors demonstrated that OMVs‐coated nanomedicine could directly interact with immune cells and activate the inflammatory response to induce cytotoxicity, thereby activating the host immune response for tumor immunotherapy. Besides, tegafur loaded in OMVs‐coated micelles could simultaneously exert chemotherapy and immune regulation, sensitizing melanoma cells to cytotoxic T lymphocytes (CTLs), further realizing remarkable inhibition of pulmonary metastasis. Therefore, in order to enrich the functions of OMVs and achieve more effective tumor suppression, two schemes have been generally adopted. The first solution is to hybridize some functionalized lipid polymers or other biological membranes to OMVs to achieve new functions or to improve their inherent performance. The second method is to take advantage of the high loading capacity of OMVs to realize the anti‐tumor immune activation of other treatment methods and OMVs per se by delivering therapeutic agents (such as chemotherapy drugs, immune adjuvants, photosensitizers, etc.) to the tumor site. Synergistic therapy could further improve the anti‐tumor efficacy. In a recent report, Qi Chen et al. designed and constructed a hybrid eukaryotic‐prokaryotic nanoplatform by fusing melanoma cytomembrane vesicles with attenuated *Salmonella* OMVs (**Figure** **12**).^[^ ^88^ ^]^ Cancer cell membrane vesicles (CCMVs) have plenty of specific antigens on the surface that can be recognized by APCs and benefit its maturation, which in turn can trigger the anti‐tumor immune response.^[^ ^89^ ^]^ Furthermore, the homologous targeting ability of CCMVs allows more vesicles to reach the tumor site,^[^ ^90^ ^]^ thus triggering a stronger immune response. In order to inherit the excellent characteristics of CCMVs and OMVs, the authors designed a fusion eukaryotic‐prokaryotic vesicle (EPV) which exhibited great potential to suppress the tumor growth based on DCs immune activation and the CTL‐derived tumor‐specific immunity. Due to the limited tumor‐eradication capacity of monoimmunotherapy, the combination with other therapeutics would be more effective. The authors made the best use of EPV's great loadability to enclose a photothermal agent inside the EPV, achieving thermal ablation of tumor mass. The immunogenic death of tumor cells caused by thermal ablation can provide supplementary antigens to the immune system and further improve the anti‐tumor immune response.

**Figure 13:**
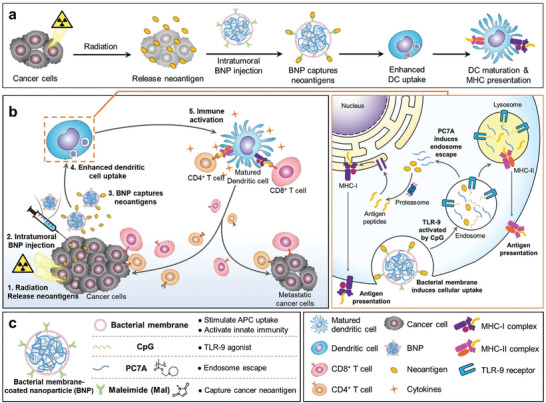
Schematic diagram of the in situ vaccine effect produced by combination of RT + BNP. a) Interaction of BNP and TME, and enhancement of APCs uptake and activation. b) Schematic description of the in situ vaccine effect initiated by combined RT + BNP. BNP could capture the neoantigen released by RT. The BNP‐neoantigen complex subsequently undergoes enhanced APCs uptake and immune activation due to the abundant PAMPs on the bacterial membrane coating of BNP. After the endocytosis of BNP, released CpG could activates TLR9, which is beneficial for APCs maturation. The mature APCs present neoantigen to CD4^+^ and CD8^+^ T cells and unregulated the secretion of pro‐inflammatory cytokines that activate antitumor immune response. c) Composition of the BNP and functions of every component. Reproduced with permission.^[^ ^91^ ^]^ Copyright 2019, Wiley‐VCH.

The superiority of membrane vehicles is not just reflected in their strong carrying capacity; the membrane can see more functions available by simply carrying out some basic modifications. For example, Ravi B. Patel and colleagues developed a bacterial membrane‐coated nanoparticle (BNP) consisting of an immunostimulator PC7A/CpG polyplex core coated with maleimide groups‐modified bacterial membrane. BNP was used as an in situ vaccine in combination with radiation therapy for cancer treatment (**Figure** **13**).^[^ ^91^ ^]^ This approach would trigger the following four sequential steps: to produce tumor‐specific antigens by radiation therapy; to capture cancer neoantigens and enhance the uptake of antigens in DCs by BNP; to initiate the anti‐tumor T‐cell response; and finally to activate both innate and adaptive immunity. Bacterial membranes are abundant in PAMPs such as TLR agonists that initiate innate immunity and DCs activation. The maleimide group modification on bacterial membranes endows a better capturability for ICD‐induced tumor‐specific antigens, thus strengthening the activation of anti‐tumor immunity. In murine melanoma or neuroblastoma, the results showed that combining BNP with radiation therapy could lead to a remarkable activation of DCs and effector T cells, significant tumor regression, and anti‐tumor immune memory, indicating that BNP has a great ability to facilitate in situ immune identification of a radiated tumor with a potent immune activation.

**Figure 14:**
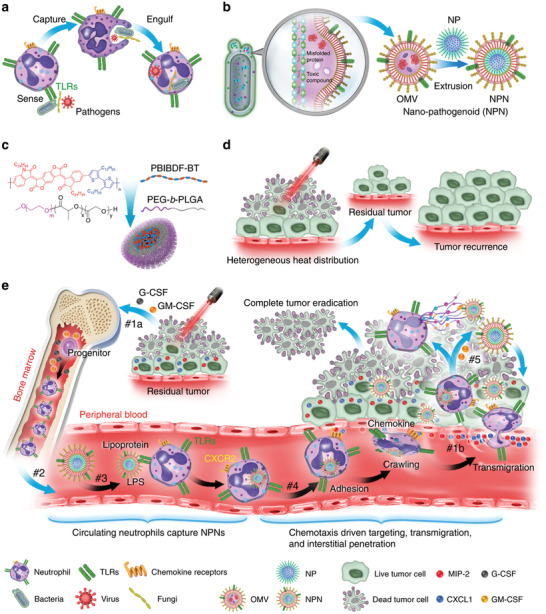
Schematic depiction of chemotaxis‐driven delivery of NPNs to eradicate tumors. a) Procedures for neutrophils to capture pathogens. b) Fabrication process of NPNs. c) Chemical structures of PEG‐*b*‐PLGA and PBIBDF‐BT. d) Insufficient penetration of laser leads to heterogeneous heat distribution within tumor mass, resulting in tumor recurrence. e) Treatment‐induced tumor cell death led to an inflammatory TME and induced the production of cytokines and chemokines. #1a The increased production of G‐CSF and GM‐CSF increased the number of neutrophils from bone marrow. #1b The increased production of CXCL1 and MIP‐2 visualized the inflamed tumor to immune system. #2 Neutrophils get into the circulating blood and encountered NPNs. #3 Neutrophils sensed NPNs with PRRs and subsequently engulfed NPNs. #4 In response to the gradient of chemokine, NPNs‐loaded neutrophils were recruited to the tumor site. #5 Released NPNs destroy tumor cells coupled with the NETs formation. Reproduced with permission.^[^ ^94^ ^]^ Copyright 2020, Springer Nature.

Apart from reaching the tumor through enhanced permeability and retention (EPR) effect, OMVs can also hitchhike the immune cells to transport them to tumor by immune response. For instance, neutrophils are important natural immune cells in mammals. On the frontline of host defender, neutrophils sense and ingest pathogens by recognizing PAMPs, followed by initiating the innate immune response to prevent the invasion of pathogenic microorganisms.^[^ ^92^ ^]^ Owing to the strong chemotactic and phagocytic function, neutrophils can quickly cross the blood vessel composed of endothelial cells (ECs) when pathogens cause infection, subsequently follow the concentration gradient of chemokines to arrive the non‐vascular area and finally migrate to the inflamed area, thus clearing the pathogens through phagocytosis and intracellular killing.^[^ ^93^ ^]^ One characteristic of tumors is inflammation, which makes neutrophils actively chemotax to tumors. Therefore, neutrophils possess natural advantages as a carrier for nanoparticles or drugs. Due to the short life cycle of neutrophils, they are extremely difficult to be engineered in vitro and injected back into the blood. An alternative solution can be the use of neutrophils and pathogens with high affinity loaded nanoparticles in the blood circulation. As a bacterial product, OMVs completely inherit the protein on the surface of the bacterial membrane. Naturally, it is also a pathogen that can be recognized by neutrophils. Min Li and colleagues designed pathogen mimicking nano‐pathogenoids (NPNs) containing PAMPs by cloaking NPs with OMVs, which can be recognized by PRRs on neutrophils (**Figure** **14**).^[^ ^94^ ^]^ After neutrophils being hitchhiked by NPNs in the blood circulation and driven by both chemokine C‐X‐C motif ligand 1 (CXCL1) and macrophage‐inflammatory protein‐2 (MIP‐2), NPNs could be carried to migrate to tumor. Then, under the stimulation of inflammation in tumor, neutrophils would release the loaded nanoparticles that were internalized by the surrounding tumor cells to kill the tumor cells. Generally speaking, OMVs are more like an efficient carrier for cancer therapeutic agents. Indeed, it showed a wide range of application scenarios in loading anticancer agents. On the other hand, more novel applications may be developed by exploiting the potential of OMVs to interact with the human immune system due to the rich and completely inherited bacterial outer membrane information on the surface of OMVs. It is promising that these applications may exert the full potential of OMVs and spawn more effective tumor immunotherapeutics.

#### 2.3.2 Bacterial Toxins

Bacterial toxins are a class of highly toxic proteins that are produced and released by bacteria with certain functions. Bacterial toxins have been proven to be a tool for cancer treatment due to high toxicity.^[^ ^95^ ^]^ The anti‐tumor bacterial toxins can be divided into two categories: conjugated on the surface antigen of tumor cells or conjugated on the ligand. Due to the high expression of specific antigens on the surface of tumor cells, bacterial toxins that specifically target these antigens, such as *Diphtheria toxin* (DT) and *Clostridium perfringens enterotoxin* (CPE), and *Pseudomonas exotoxin* (PE) can be used for targeting and killing cancer cells.^[^ ^96^ ^]^ Among them, DT has been widely used in tumor treatment both in mice and humans because of its relatively desiable anti‐tumor effect.^[^ ^97^ ^]^ This may benefit from its strong cytotoxicity or the simultaneously induced anti‐tumor immunity. Silvio Buzzi and colleagues employed a non‐toxic cross‐reacting material 197 (CRM197) to treat a group of cancer patients.^[^ ^98^ ^]^ CRM197 is a nontoxic mutant of DT that possesses similar immunological properties as DT. Similar to DT, CRM197 targets heparin‐binding epidermal growth factor (HB‐EGF), which is commonly overexpressed in cancer cells. The authors found that subcutaneous injection of CRM197 caused inflammatory‐immunological reactions, triggering biological anti‐tumor response. Through the experimental results, the authors speculated that neutrophils and TNF‐*α* were involved in the anti‐tumor process. Thus, bacterial toxins not only show a great lethal effect on cancer cells but also initiate anti‐tumor immunity. The fusion proteins composed of bacterial toxins and targeting antibody fragments are also called immunotoxins.^[^ ^99^ ^]^ The fused targeting antibody fragment can target cancer cells, leading to a more potent lethal effect of bacterial toxin fragment on target cells. Immunotoxins that contain bacterial toxins which achieve potent cytotoxicity by blocking protein translation have been proved to be effective in treating some hematological malignancies.^[^ ^100^ ^]^ In a previous study, Ontak, a fusion protein composed of DT and anti‐IL‐2, was studied for treating chronic lymphocytic leukaemia (CLL) based on the overexpression of high‐affinity IL‐2 receptors on CLL cells, and satisfactory results were achieved.^[^ ^101^ ^]^ Similar to chemotherapy, repeated administration is necessary for immunotoxins treatment to maintain the optimal cell lethal concentration. However, the retreatment is limited to its immunogenetics, which is also known as the formation of anti‐drug antibodies (ADA). After treatment with immunotoxin, many patients will have a rapid immune response and the formation of ADA, which will neutralize the efficacy of immunotoxins and prohibit multiple administrations. To solve this problem, some researchers have tried to combine immunotoxin with chemo drugs or modify bacterial toxins to evade recognition of the immune system. These methods have achieved effective immunogenetic reduction. A more direct and well‐accepted method is to delete or mutate T cell epitopes by rational design of recombinant proteins to achieve immunogenetic reduction. Ronit Mazor et al. reported a novel immunotoxin consisting of disulfide stabilized Fv of anti‐Tac antibody and PE38 with nine point mutations both in domains II and III. Moreover, they demostrated that domain II is essential to CD25‐mediated cell killing, which is different from CD22‐mediated internalization.^[^ ^102^ ^]^ Compared with LMB‐2 (anti‐Tac(Fv)‐PE38), the newly constructed immunotoxin LMB‐142 shows both a strong cytotoxic activity in vitro and a five‐time lower nonspecific toxicity in mice.

In addition to using immunotoxins to treat T‐cell malignancies, Tregs depleting fusion protein toxins create a paradigm shift in cancer immunotherapy for other solid tumors as well. Tregs are an important population of T cells, which are regarded as the brake of the effector T cell‐mediated immune response. Besides, Tregs play important roles in immune tolerance, prevention of autoimmune diseases, and suppression of anti‐tumor immunity.^[^ ^103^ ^]^ As the vanguard of the immunosuppressive microenvironment, Tregs promote the growth of tumors. This has prompted researchers to develop many immunotherapies for Tregs, including their function suppression and consumption.^[^ ^104^ ^]^ One method of depleting Tregs is to reposition bacterial toxins to take advantage of their efficient cytotoxicity to kill Tregs directly. This approach involves replacing the natural binding domain of bacterial toxins with known ligands of Treg receptors, transferring the killing mechanism of toxins to the cells rich in Treg receptors, thereby eliminating them instead. Benefits include the alleviation of immunosuppression of TME and the minimization of the toxicity associated with bacterial toxins to non‐target cells.

The high expression of Foxp3 in Tregs leads to a high‐level CD25 expression on the surface of Tregs, forming a heterotrimeric high‐affinity IL‐2 receptors.^[^ ^105^ ^]^ Studies have shown that the abundant CD25 on Tregs can consume IL‐2 in the local environment, while the lack of cytokines will cause the apoptosis of activated effector T cells.^[^ ^106^ ^]^ Therefore, CD25 can be selected as an ideal targeting site. Laurene S. Cheung and co‐workers developed a new generation of IL‐2 receptor‐targeted diphtheria fusion toxin, which has a good anti‐tumor effect related to the reduction of Tregs. In fact, this new‐generation fusion toxin has also seen a good synergy effect together with anti‐PD‐1 to treat melanoma.^[^ ^107^ ^]^ The risk of vascular leakage and production issues (e.g., purity and aggregation) limit the clinical application of *denileukin diftitox* (Ontak), which is a fusion protein composed of the bacterial toxin DT and anti‐IL‐2. The authors reported a production method that employed Corynebacterium diphtheriae to directly replicate biologically‐active and fully‐folded s‐DAB‐IL‐2 as a monomer into the culture medium. Furthermore, the authors prepared a more advanced fusion protein s‐DAB‐IL‐2(V6A) with a single amino acid mutation (V6A). Compared with s‐DAB‐IL‐2, V6A reduced vascular leak in vitro by 50× and lethality in mice by 3.7×. In mouse melanoma model, significant suppression in tumor growth was observed for both s‐DAB‐IL‐2(V6A) monotherapy and the combination therapy with anti‐PD‐1. The authors analyzed and confirmed that the excellent therapeutic effect was related to the depletion of Tregs and the enhancement of effector T cells.

Although bacterial toxins have been widely studied on imposing the great toxicity on tumor cells for directly killing, it is also an effective method for fusion with Tregs targeted proteins for Tregs depletion and anti‐tumor response enhancement. More hopefully, the successful combination with immune checkpoint blockade makes it possible for bacterial toxins to be applied in cancer immunotherapy.

#### 2.3.3 Bacterial Spores

**Figure 15:**
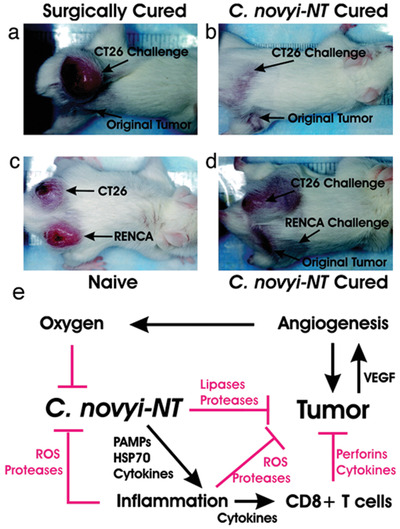
Photographs of tumor bearing mice used in challenge experiments. a) The surgically cured CT26 tumor bearing mouse was not resistant to the re‐challenge of CT26 cells. b) The mouse cured with *C. novyi‐NT* treatment was resistant to the re‐challenge. c) CT26 tumor and RENCA tumor grew in naive mice post inoculation of corresponding cells. d) When mice treated and cured with *C. novyi‐NT* of their RENCA tumors were similarly re‐challenged, only CT26 tumors formed. e) Working mechanism flow chart. *C. novyi‐NT* spores colonized in hypoxic/anoxic core area of tumors. Then they germinate and cause lysis of tumor cells. The tumor cell lysate elicit inflammatory responses, as well as the raised oxygen concentration in nearby areas, inhibits the proliferation and spread of *C. novyi‐NT*. *C. novyi‐NT*‐induced inflammation also promotes tumor destruction rely on the production of ROS, proteases, perforins, and tumoricidal cytokines. Also, the inflammatory response activates specific cellular antitumor immune responses that suppress tumor growth. Reproduced with permission.^[^ ^25a^ ^]^ Copyright 2004, National Academy of Sciences.

As a dormant form of bacteria, spores are highly resistant.^[^ ^108^ ^]^ They can survive in oxygen‐rich tissues for a long time without germination. When encountering a suitable environment, such as the hypoxic/necrotic area of the tumor core, spores will germinate and multiply. There is no severe hypoxic environment in normal human tissues, which means that spores will not show toxicity to normal human organs. Researchers have tried to inject *Clostridium histolyticum* spore suspension into the tumor and observed effective suppression of transplanted mouse sarcomas without obvious systemic toxicity.^[^ ^97^ ^]^ Not only limited to intratumoral injection, some researchers observed the death of mice due to tetanus within 48 h after intravascular injection of *Clostridium* spores in tumor‐bearing mice.^[^ ^109^ ^]^ The healthy mice with the same treatment were asymptomatic for 40 days. This confirms that spores exhibit tumor‐specific germination even with vascular administration. *Clostridium novyi* (*C. novyi*) has been widely investigated because of its extreme sensitivity to oxygen and its excellent mobility owing to the numerous peritrichous flagella.^[^ ^24c^ ^]^ These two characteristics lead to tumor enrichment of *C. novyi* even with only a small amount of spore germination. Considering the toxicity, the major systemic toxin (*α*‐toxin) gene of *C. novyi* was removed, and a new attenuated *C. novyi‐NT* was created which has a better prospect for application because of its lower systemic toxicity.^[^ ^110^ ^]^ Nishant Agrawal et al. observed that systemic injection of *C.novyi‐NT* spores in fully immune tumor‐bearing mice could achieve tumor regression and have long‐term effects (**Figure** **15**).^[^ ^25a^ ^]^ The authors speculated based on the experimental results that *C. novyi‐NT* spores spread all over the body after the systemic injection. However, the strict anaerobic properties made them only germinate in the necrotic core of tumor, which is significantly hypoxic. The germinated bacteria will kill nearby tumor cells by locally secreting lipases, proteases and other degrading enzymes. In the meanwhile, the host responds to this local infection and produces immune‐promoting cytokines, such as IL‐6, MIP‐2, granulocyte colony‐stimulating factor (G‐CSF) and tissue inhibitor of metalloproteinases 1 (TIMP‐1) to attract tumoral infiltration of various immune cells. At the very beginning, it was mainly a neutrophil response, but led to the participation of monocytes subsequently. The inflammatory response can inhibit the spread of bacterial infections and provide a second layer of control besides the first layer provided by an anaerobic environment. Inflammation may also directly cause tumor cells’ destruction through the production of ROS, proteases and other enzymes. Moreover, inflamation induces an effective cellular immune response, which continues to destroy the remained tumor cells that have not been killed by the bacteria. The authors observed that 30% of tumor‐bearing mice were cured, which is a quite gratifying result. In a later report, injection of *C. novyi‐NT* spores into the naturally occurring tumors on dogs induced a strong immune response.^[^ ^111^ ^]^ The authors observed that intratumoral administration of *C. novyi‐NT* spores resulted in enhanced phagocytosis and increased NK cell‐like function. Intravenous injection of *C. novyi‐NT* spores will lead to LPS‐triggered TNF‐*α* production, LTA‐triggered IL‐10 production, and also increasing of NK cell‐like function. This indicates that the administration of *C. novyi‐NT* spores can induce long‐term changes in immune cell function. In another report, John T. Heaps and colleagues injected engineered Clostridial spores into blood vessels to effectively suppress and cure human colon carcinoma in the mouse xenograft model.^[^ ^112^ ^]^ The engineered spores can germinate and be activated after reaching the hypoxic necrosis area of the tumor core, and then release the prodrug‐converting enzyme (PCE) that can convert non‐toxic prodrug molecule into a strong cytotoxic form at the tumor site, a process further causing tumor cells into death.

## 3 Conclusion

Further clarifications on the interaction between tumor and the human immune system has unveiled that immunotherapy is one of the most promising approaches to cure cancer.^[^ ^113^ ^]^ Since both bacteria and their components display inherent stimulation to the host immune system, thereby producing a positive anti‐tumor immune response, the bacteria‐based tumor immunotherapy has developed rapidly. From bacteria attenuation, transformation engineering, and individual component extraction and application to synergistic therapy, researchers have tried a variety of solutions to obtain the optimal efficacy. The latest research also shows that bacteria play a vital role in both the occurrence and development of tumors.^[^ ^114^ ^]^ Although the triangle interactions between bacteria, tumors, and the immune system are not yet clear, it is hopeful that with the deepening of research, researchers can make the best use of the great adaptability of bacteria to regulate the interaction of the other two to achieve better efficacy.

Current engineered bacteria exhibit new functions that enable them to locally produce and release immune checkpoint blockers or other immunotherapeutics in tumors.^[^ ^42^, ^46^, ^61^ ^]^ Due to the specific colonization of bacteria in the tumor, the chance of immunotherapeutics reaching the target is greatly improved, thus reducing the repeated administration of free drugs and reducing the possible side effects. However, bacteria are complex therapeutic agents with vitality rather than precise formulations. The targeting effect of bacteria is variable, and there may be differences in efficacy between patients with different tumors during treatment. Besides, the level of immunotherapeutics expressed by bacteria may vary. Too low expression levels may result in poor efficacy, while excessive expression levels may cause autoimmune diseases. The long‐term stable production of bacteria in the host is very important. Moreover, some uncontrollable mutations may bring toxicity to patients in the process of bacteria proliferation. Fortunately, with the rapid development of synthetic biology, solutions to these potential problems may be available and worth of exploration. Researchers can precisely control the production and release behavior of immunotherapeutics by controlling gene copy number, promoter strength, bacterial metabolic rate, and initial bacteria injection dose. For patients with different tumors and at different stages, a personalized plan is preferred by adjusting the expression intensity of the therapeutics to yield the optimized treatment effect.

Although the administration of bacteria into the body can cause a variety of immune cell responses to produce anti‐tumor efficacy, most of the bacteria are colonized in the core necrosis area of the tumor with less distribution in the outermost area of the tumor.^[^ ^115^ ^]^ Therefore, the immune cells infiltrated in the tumor may have spatially distributed heterogeneity, resulting in an unsatisfactory anti‐tumor effect. Some other tumor treatment methods (such as radiotherapy) have a better therapeutic effect on areas where the outer layer of the tumor has good oxygen perfusion. The combination of these therapies focusing on different areas may produce better treatment results.

Some extracted components of bacteria are used as immunostimulators. Although they can effectively elicit anti‐tumor immune activation, their single action site in the entire anti‐tumor immune response link extremely limits the maximum efficacy. Despite that the increased dose or rounds of injection can better suppress tumor growth, some new problems come out. One of the problems is that the extraction of certain components of bacteria with high purity is a very time‐consuming task and may limit large‐scale applications. Another problem is that patients may not accept multiple injections as easily as oral administration. Therefore, replacement of oral dosage forms will help patients better accept multiple administrations. As a promising treatment approach, oral administration has attracted increasingly more attention in recent years not only because of the most natural and easiest administration, but also its self‐assistance that eliminates the requirement for nursing. However, it still remains a challenge as the complex physiological conditions (e.g., strong acid condition in stomach, presence of different enzymes) in gastrointestinal tract may digest or deactivate drugs or bacteria before they reach the action site. In order to avoid these problems, the therapeutics is usually wrapped in some protective materials and made into capsules to improve the utilization rate and also achieve sustained release. As another alternative and creative strategy, some researchers took full advantage of bioengineering technology to retrofit orally‐taken bacteria to serve as “living factory” to produce “products” in the gut, which may play beneficial roles in microbiome‐induced anti‐tumor immunity. Different from tumor‐targeting bacteria, orally‐taken bacteria prefer probiotics (such as *Lactobacillus* and *Bifidobacterium* etc.) to pathogenic bacteria.^[^ ^116^ ^]^ Meanwhile, most probiotics are less virulent than pathogenic bacteria, even compared with attenuated strains, which completely eliminates the worry of potential toxicity on patients.

The complexity of bacteria as a living body determines the difficulties and risks of transforming them into weapons to fight against tumor. However, complexity is precisely the most fascinating part of bacteria, which enables scientist to fine‐tune the various functions of different strains to realize anti‐tumor activities unachievable via other therapies. This complexity is also reflected in the interactions between bacteria and the human immune system. On top of that, with the in‐depth understanding of their relationship, bacteria‐based cancer immunotherapy will provide a steady stream of power to fight against cancer.

## Conflict of Interest

The authors declare no conflict of interest.

## References

[1] a) J. C. Forster , W. M. Harriss‐Phillips , M. J. Douglass , E. Bezak , Hypoxia 2017, 5, 21; 28443291 10.2147/HP.S133231 PMC5395278

[2] a) K. Palucka , J. Banchereau , Nat. Rev. Cancer 2012, 12, 265; 22437871 10.1038/nrc3258 PMC3433802

[3] a) R. Kim , Cancer Immunotherapy, Academic Press, San Diego, CA 2007, p. 9;

[4] a) C. West , F. Slevin , Clin. Oncol. 2019, 31, 595; 10.1016/j.clon.2019.06.008 31301956

[5] a) X. Huang , J. Zhuang , S. W. Chung , B. Huang , G. Halpert , K. Negron , X. Sun , J. Yang , Y. Oh , P. M. Hwang , ACS Nano 2018, 13, 236; 30576113 10.1021/acsnano.8b05399 PMC8323471

[6] a) S. Wilhelm , A. J. Tavares , Q. Dai , S. Ohta , J. Audet , H. F. Dvorak , W. C. Chan , Nat. Rev. Mater. 2016, 1, 16014;

[7] N. S. Forbes , Nat. Rev. Cancer 2010, 10, 785. 20944664 10.1038/nrc2934 PMC3756932

[8] W. B. Coley , Ann. Surg. 1891, 14, 199. 10.1097/00000658-189112000-00015 PMC1428624 17859590

[9] S. i. Taniguchi , M. Fujimori , T. Sasaki , H. Tsutsui , Y. Shimatani , K. Seki , J. Amano , Cancer Sci. 2010, 101, 1925. 20579076 10.1111/j.1349-7006.2010.01628.x PMC11158574

[10] N. S. Forbes , L. L. Munn , D. Fukumura , R. K. Jain , Cancer Res. 2003, 63, 5188. 14500342

[11] S. Leschner , K. Westphal , N. Dietrich , N. Viegas , J. Jablonska , M. Lyszkiewicz , S. Lienenklaus , W. Falk , N. Gekara , H. Loessner , PLoS One 2009, 4, e6692. 19693266 10.1371/journal.pone.0006692 PMC2724709

[12] N. Bernardes , A. M. Chakrabarty , A. M. Fialho , Appl. Microbiol. Biotechnol. 2013, 97, 5189. 23644748 10.1007/s00253-013-4926-6

[13] a) L. Zitvogel , M. Ayyoub , B. Routy , G. Kroemer , Cell 2016, 165, 276;. 27058662 10.1016/j.cell.2016.03.001

[14] M. Triantafilou , K. Triantafilou , Trends Immunol. 2002, 23, 301. 12072369 10.1016/s1471-4906(02)02233-0

[15] a) L. Samavati , R. Rastogi , W. Du , M. Hüttemann , A. Fite , L. Franchi , Mol. Immunol. 2009, 46, 1867;. 19299019 10.1016/j.molimm.2009.02.018

[16] L. Sfondrini , A. Rossini , D. Besusso , A. Merlo , E. Tagliabue , S. Mènard , A. Balsari , J. Immunol. 2006, 176, 6624. 16709820 10.4049/jimmunol.176.11.6624

[17] S. Dasari , C. Kathera , A. Janardhan , A. P. Kumar , B. Viswanath , Clin. Nutr. 2017, 36, 1465. 27923508 10.1016/j.clnu.2016.11.017

[18] J. Ahn , H. Kim , K. M. Yang , J. Microbiol. Biotechnol. 2020, 30, 313. 32238757 10.4014/jmb.2003.03011 PMC9728410

[19] H. C. Nauts , W. E. Swift , B. L. Coley , Cancer Res. 1946, 6, 205. 21018724

[20] a) M. Cronin , R. M. Stanton , K. P. Francis , M. Tangney , Cancer Gene Ther. 2012, 19, 731;. 22996740 10.1038/cgt.2012.59

[21] C. H. Lee , Appl. Microbiol. Biotechnol. 2012, 93, 517. 22120621 10.1007/s00253-011-3695-3

[22] C. Clairmont , K. C. Lee , J. Pike , M. Ittensohn , K. B. Low , J. Pawelek , D. Bermudes , S. M. Brecher , D. Margitich , J. Turnier , Z. Li , X. Luo , I. King , L. M. Zheng , J. Infect. Dis. 2000, 181, 1996. 10837181 10.1086/315497

[23] a) D.‐W. Zheng , Y. Chen , Z.‐H. Li , L. Xu , C.‐X. Li , B. Li , J.‐X. Fan , S.‐X. Cheng , X.‐Z. Zhang , Nat. Commun. 2018, 9, 1680; 29700283 10.1038/s41467-018-03233-9 PMC5920064

[24] a) R. W. Kasinskas , N. S. Forbes , Biotechnol. Bioeng. 2006, 94, 710; 16470601 10.1002/bit.20883

[25] a) N. Agrawal , C. Bettegowda , I. Cheong , J. F. Geschwind , C. G. Drake , E. L. Hipkiss , M. Tatsumi , L. H. Dang , L. A. Diaz , M. Pomper , M. Abusedera , R. L. Wahl , K. W. Kinzler , S. B. Zhou , D. L. Huso , B. Vogelstein , Proc. Natl. Acad. Sci. U. S. A. 2004, 101, 15172; . 15471990 10.1073/pnas.0406242101 PMC523456

[26] S. H. Kim , F. Castro , Y. Paterson , C. Gravekamp , Cancer Res. 2009, 69, 5860. 19584282 10.1158/0008-5472.CAN-08-4855 PMC3127451

[27] M. P. Brynildsen , J. A. Winkler , C. S. Spina , I. C. MacDonald , J. J. Collins , Nat. Biotechnol. 2013, 31, 160. 23292609 10.1038/nbt.2458 PMC3568245

[28] D. Chandra , A. Jahangir , W. Quispe‐Tintaya , M. Einstein , C. Gravekamp , Br. J. Cancer 2013, 108, 2281. 23640395 10.1038/bjc.2013.206 PMC3681012

[29] a) C. Bettegowda , X. Huang , J. Lin , I. Cheong , M. Kohli , S. A. Szabo , X. Zhang , L. A. Diaz , V. E. Velculescu , G. Parmigiani , Nat. Biotechnol. 2006, 24, 1573; 17115055 10.1038/nbt1256 PMC9338427

[30] M. Shinnoh , M. Horinaka , T. Yasuda , S. Yoshikawa , M. Morita , T. Yamada , T. Miki , T. Sakai , Int. J. Oncol. 2013, 42, 903. 23354042 10.3892/ijo.2013.1790

[31] Y. Guo , Y. Chen , X. Liu , J.‐J. Min , W. Tan , J. H. Zheng , Cancer Lett. 2020, 469, 102. 31666180 10.1016/j.canlet.2019.10.033

[32] a) S. Zhou , Z. Zhao , Y. Lin , S. Gong , F. Li , J. Pan , X. Li , Z. Gao , A. Z. Zhao , Cancer Biol. Ther. 2016, 17, 732; 27089121 10.1080/15384047.2016.1177683 PMC4970537

[33] T. X. Phan , V. H. Nguyen , M. T. Q. Duong , Y. Hong , H. E. Choy , J. J. Min , Microbiol. Immunol. 2015, 59, 664. 26500022 10.1111/1348-0421.12333

[34] a) S. Felgner , D. Kocijancic , M. Frahm , U. Heise , M. Rohde , K. Zimmermann , C. Falk , M. Erhardt , S. Weiss , Oncoimmunology 2018, 7, e1382791;. 29308303 10.1080/2162402X.2017.1382791 PMC5749626

[35] A. Kupz , R. Curtiss III , S. Bedoui , R. A. Strugnell , PLoS One 2014, 9, e97418. 24827856 10.1371/journal.pone.0097418 PMC4020851

[36] a) F. Saccheri , C. Pozzi , F. Avogadri , S. Barozzi , M. Faretta , P. Fusi , M. Rescigno , Sci. Transl. Med. 2010, 2, 44ra57; . 10.1126/scitranslmed.3000739 20702856

[37] Y. Ma , R. Conforti , L. Aymeric , C. Locher , O. Kepp , G. Kroemer , L. Zitvogel , Cancer Metastasis Rev. 2011, 30, 71. 21298323 10.1007/s10555-011-9283-2

[38] J. F. Toso , V. J. Gill , P. Hwu , F. M. Marincola , N. P. Restifo , D. J. Schwartzentruber , R. M. Sherry , S. L. Topalian , J. C. Yang , F. Stock , J. Clin. Oncol. 2002, 20, 142. 11773163 10.1200/JCO.2002.20.1.142 PMC2064865

[39] S. Patyar , R. Joshi , D. P. Byrav , A. Prakash , B. Medhi , B. Das , J. Biomed. Sci. 2010, 17, 21. 20331869 10.1186/1423-0127-17-21 PMC2854109

[40] a) D. L. Mager , J. Transl. Med. 2006, 4, 14;. 16566840 10.1186/1479-5876-4-14 PMC1479838

[41] a) S. Alizadeh , A. Esmaeili , A. Barzegari , M. A. Rafi , Y. Omidi , J. Drug Target. 2020, 28, 700;. 32116051 10.1080/1061186X.2020.1737087

[42] S. Chowdhury , S. Castro , C. Coker , T. E. Hinchliffe , N. Arpaia , T. Danino , Nat. Med. 2019, 25, 1057. 31270504 10.1038/s41591-019-0498-z PMC6688650

[43] M. O. Din , T. Danino , A. Prindle , M. Skalak , J. Selimkhanov , K. Allen , E. Julio , E. Atolia , L. S. Tsimring , S. N. Bhatia , J. Hasty , Nature 2016, 536, 81. 27437587 10.1038/nature18930 PMC5048415

[44] J. Xu , R. Saklatvala , S. Mittal , S. Deshmukh , A. Procopio , Adv. Sci. 2020, 7, 1903394. 10.1002/advs.201903394 PMC7175294 32328428

[45] a) M. A. Postow , R. Sidlow , M. D. Hellmann , N. Engl. J. Med. 2018, 378, 158; 29320654 10.1056/NEJMra1703481

[46] C. R. Gurbatri , I. Lia , R. Vincent , C. Coker , S. Castro , P. M. Treuting , T. E. Hinchliffe , N. Arpaia , T. Danino , Sci. Transl. Med. 2020, 12, eaax0876. 32051224 10.1126/scitranslmed.aax0876 PMC7685004

[47] C. J. Jones , S.‐I. Aizawa , Advances in Microbial Physiology, Vol. 32, Academic Press, San Diego, CA 1991, p. 109. 1882727 10.1016/s0065-2911(08)60007-7

[48] K. Namba , F. Vonderviszt , Q. Rev. Biophys. 1997, 30, 1. 9134575 10.1017/s0033583596003319

[49] A. M. Stadler , T. Unruh , K. Namba , F. Samatey , G. Zaccai , Biophys. J. 2013, 105, 2157. 24209861 10.1016/j.bpj.2013.09.039 PMC3824299

[50] a) M. Vijay‐Kumar , A. Gewirtz , Mucosal Immunol. 2009, 2, 197; 19242410 10.1038/mi.2009.9

[51] T. K. Means , F. Hayashi , K. D. Smith , A. Aderem , A. D. Luster , J. Immunol. 2003, 170, 5165. 12734364 10.4049/jimmunol.170.10.5165

[52] S. E. Lee , S. H. Hong , V. Verma , Y. S. Lee , T.‐M. N. Duong , K. Jeong , S. Uthaman , Y. C. Sung , J.‐T. Lee , I.‐K. Park , OncoImmunology 2016, 5, e1081328. 27057462 10.1080/2162402X.2015.1081328 PMC4801456

[53] a) M. Vijay‐Kumar , J. D. Aitken , A. Kumar , A. S. Neish , S. Uematsu , S. Akira , A. T. Gewirtz , Infect. Immun. 2008, 76, 1276;. 18195036 10.1128/IAI.01491-07 PMC2258833

[54] X. Yu , C. Guo , H. Yi , J. Qian , P. B. Fisher , J. R. Subjeck , X.‐Y. Wang , Cancer Res. 2013, 73, 2093. 23333935 10.1158/0008-5472.CAN-12-1740 PMC3618619

[55] D. Geng , S. Kaczanowska , A. Tsai , K. Younger , A. Ochoa , A. P. Rapoport , S. Ostrand‐Rosenberg , E. Davila , Cancer Res. 2015, 75, 1959. 25795705 10.1158/0008-5472.CAN-14-2467 PMC4433615

[56] A. Mantovani , S. Sozzani , M. Locati , P. Allavena , A. Sica , Trends Immunol. 2002, 23, 549. 12401408 10.1016/s1471-4906(02)02302-5

[57] J. H. Zheng , V. H. Nguyen , S. N. Jiang , S. H. Park , W. Tan , S. H. Hong , M. G. Shin , I. J. Chung , Y. Hong , H. S. Bom , H. E. Choy , S. E. Lee , J. H. Rhee , J. J. Min , Sci. Transl. Med. 2017, 9, eaak9537. 28179508 10.1126/scitranslmed.aak9537

[58] a) H. Ishikawa , Z. Ma , G. N. Barber , Nature 2009, 461, 788; 19776740 10.1038/nature08476 PMC4664154

[59] a) J. Fu , D. B. Kanne , M. Leong , L. H. Glickman , S. M. McWhirter , E. Lemmens , K. Mechette , J. J. Leong , P. Lauer , W. Liu , Sci. Transl. Med. 2015, 7, 283ra52; . 10.1126/scitranslmed.aaa4306 PMC4504692 25877890

[60] B. Larkin , V. Ilyukha , M. Sorokin , A. Buzdin , E. Vannier , A. Poltorak , J. Immunol. 2017, 199, 397. 28615418 10.4049/jimmunol.1601999 PMC5525333

[61] D. S. Leventhal , A. Sokolovska , N. Li , C. Plescia , S. A. Kolodziej , C. W. Gallant , R. Christmas , J.‐R. Gao , M. J. James , A. Abin‐Fuentes , Nat. Commun. 2020, 11, 2739. 32483165 10.1038/s41467-020-16602-0 PMC7264239

[62] M. Loeffler , G. Le'Negrate , M. Krajewska , J. C. Reed , Proc. Natl. Acad. Sci. U. S. A. 2007, 104, 12879. 17652173 10.1073/pnas.0701959104 PMC1937560

[63] M. Loeffler , G. Le'Negrate , M. Krajewska , J. C. Reed , Cancer Gene Ther. 2008, 15, 787. 18654612 10.1038/cgt.2008.48 PMC2760299

[64] a) X. Guo , X. Wei , Z. Chen , X. Zhang , G. Yang , S. Zhou , Prog. Mater. Sci. 2020, 107, 100599;

[65] F. Xu , X. Huang , Y. Wang , S. Zhou , Adv. Mater. 2020, 32, 1906745. 10.1002/adma.201906745 32105374

[66] X. Huang , F. Xu , H. Hou , J. Hou , Y. Wang , S. Zhou , Nano Res. 2019, 12, 1361.

[67] H. Hou , X. Huang , G. Wei , F. Xu , Y. Wang , S. Zhou , ACS Appl. Mater. Interfaces 2019, 11, 29579. 31359756 10.1021/acsami.9b09671

[68] J. E. Cohen , A. B. Goldstone , M. J. Paulsen , Y. Shudo , A. N. Steele , B. B. Edwards , J. B. Patel , J. W. MacArthur , M. S. Hopkins , C. E. Burnett , Sci. Adv. 2017, 3, e1603078. 28630913 10.1126/sciadv.1603078 PMC5470824

[69] L. Liu , H. He , Z. Luo , H. Zhou , R. Liang , H. Pan , Y. Ma , L. Cai , Adv. Funct. Mater. 2020, 30, 1910176.

[70] L. Galluzzi , A. Buqué , O. Kepp , L. Zitvogel , G. Kroemer , Nat. Rev. Immunol. 2017, 17, 97. 27748397 10.1038/nri.2016.107

[71] J. Fan , H. Cai , Q. Li , Z. Du , W. Tan , J. Biotechnol. 2012, 158, 104. 22306110 10.1016/j.jbiotec.2012.01.017

[72] S. M. Hatfield , J. Kjaergaard , D. Lukashev , T. H. Schreiber , B. Belikoff , R. Abbott , S. Sethumadhavan , P. Philbrook , K. Ko , R. Cannici , Sci. Transl. Med. 2015, 7, 277ra30. 10.1126/scitranslmed.aaa1260 PMC4641038 25739764

[73] A. Facciabene , X. Peng , I. S. Hagemann , K. Balint , A. Barchetti , L.‐P. Wang , P. A. Gimotty , C. B. Gilks , P. Lal , L. Zhang , Nature 2011, 475, 226. 21753853 10.1038/nature10169

[74] H. Chen , A. Adam , Y. Cheng , S. Tang , J. Hartung , E. Bao , Mol. Med. Rep. 2015, 11, 2276. 25412361 10.3892/mmr.2014.2986

[75] W. Chen , Y. Wang , M. Qin , X. Zhang , Z. Zhang , X. Sun , Z. Gu , ACS Nano 2018, 12, 5995. 29786420 10.1021/acsnano.8b02235

[76] W. Chen , Z. Guo , Y. Zhu , N. Qiao , Z. Zhang , X. Sun , Adv. Funct. Mater. 2019, 30, 1906623.

[77] P. Sasikumar , R. Shrimali , S. Adurthi , R. Ramachandra , L. Satyam , A. Dhudashiya , D. Samiulla , K. Sunilkumar , M. Ramachandra , J. ImmunoTher. Cancer 2013, 1, O24.

[78] T. A. Kufer , Bacterial Membrane Vesicles: Biogenesis, Functions and Applications, Springer Nature, Berlin 2020.

[79] a) M. Kaparakis‐Liaskos , R. L. Ferrero , Nat. Rev. Immunol. 2015, 15, 375; 25976515 10.1038/nri3837

[80] a) A. T. Jan , Front. Microbiol. 2017, 8, 1053; 28649237 10.3389/fmicb.2017.01053 PMC5465292

[81] S. Roier , F. G. Zingl , F. Cakar , S. Durakovic , P. Kohl , T. O. Eichmann , L. Klug , B. Gadermaier , K. Weinzerl , R. Prassl , Nat. Commun. 2016, 7, 10515. 26806181 10.1038/ncomms10515 PMC4737802

[82] C. Schwechheimer , D. L. Rodriguez , M. J. Kuehn , MicrobiologyOpen 2015, 4, 375. 25755088 10.1002/mbo3.244 PMC4475382

[83] C. Schwechheimer , A. Kulp , M. J. Kuehn , BMC Microbiol. 2014, 14, 324. 25528573 10.1186/s12866-014-0324-1 PMC4302634

[84] a) V. Gujrati , S. Kim , S.‐H. Kim , J. J. Min , H. E. Choy , S. C. Kim , S. Jon , ACS Nano 2014, 8, 1525; 24410085 10.1021/nn405724x

[85] S. Delannoy , L. Beutin , P. Mariani‐Kurkdjian , A. Fleiss , S. Bonacorsi , P. Fach , Front. Cell. Infect. Microbiol. 2017, 7, 30. 28224115 10.3389/fcimb.2017.00030 PMC5293828

[86] a) W. Song , A. C. Anselmo , L. Huang , Nat. Nanotechnol. 2019, 14, 1093; 31802032 10.1038/s41565-019-0589-5

[87] Q. Chen , H. Bai , W. Wu , G. Huang , Y. Li , M. Wu , G. Tang , Y. Ping , Nano Lett. 2020, 20, 11. 31858807 10.1021/acs.nanolett.9b02182

[88] Q. Chen , G. Huang , W. Wu , J. Wang , J. Hu , J. Mao , P. K. Chu , H. Bai , G. Tang , Adv. Mater. 2020, 32, 1908185. 10.1002/adma.201908185 32108390

[89] R. H. Fang , C.‐M. J. Hu , B. T. Luk , W. Gao , J. A. Copp , Y. Tai , D. E. O'Connor , L. Zhang , Nano Lett. 2014, 14, 2181. 24673373 10.1021/nl500618u PMC3985711

[90] a) Z. Chen , P. Zhao , Z. Luo , M. Zheng , H. Tian , P. Gong , G. Gao , H. Pan , L. Liu , A. Ma , ACS Nano 2016, 10, 10049; . 27934074 10.1021/acsnano.6b04695

[91] R. B. Patel , M. Ye , P. M. Carlson , A. Jaquish , L. Zangl , B. Ma , Y. Wang , I. Arthur , R. Xie , R. J. Brown , X. Wang , R. Sriramaneni , K. Kim , S. Gong , Z. S. Morris , Adv. Mater. 2019, 31, 1902626. 10.1002/adma.201902626 PMC6810793 31523868

[92] A. Chalifour , P. Jeannin , J.‐F. Gauchat , A. Blaecke , M. Malissard , T. N'Guyen , N. Thieblemont , Y. Delneste , Blood 2004, 104, 1778. 15166032 10.1182/blood-2003-08-2820

[93] E. Kolaczkowska , P. Kubes , Nat. Rev. Immunol. 2013, 13, 159. 23435331 10.1038/nri3399

[94] M. Li , S. Li , H. Zhou , X. Tang , Y. Wu , W. Jiang , Z. Tian , X. Zhou , X. Yang , Y. Wang , Nat. Commun. 2020, 11, 1126. 32111847 10.1038/s41467-020-14963-0 PMC7048836

[95] A. J. Lax , Nat. Rev. Microbiol. 2005, 3, 343. 15806096 10.1038/nrmicro1130

[96] a) F. Shafiee , M. G. Aucoin , A. Jahanian Najafabadi , Front. Microbiol. 2019, 10, 2340; 31681205 10.3389/fmicb.2019.02340 PMC6813239

[97] L. R. Weerakkody , C. Witharana , Life Sci. 2019, 235, 116839. 31499068 10.1016/j.lfs.2019.116839

[98] S. Buzzi , D. Rubboli , G. Buzzi , A. M. Buzzi , C. Morisi , F. Pironi , Cancer Immunol. Immunother. 2004, 53, 1041. 15168087 10.1007/s00262-004-0546-4 PMC11032786

[99] I. Pastan , M. C. Willingham , D. J. FitzGerald , Cell 1986, 47, 641. 3536124 10.1016/0092-8674(86)90506-4

[100] a) S. McCann , O. E. Akilov , L. Geskin , Clin. J. Oncol. Nurs. 2012, 16, E164; 23022942 10.1188/12.CJON.E164-E172

[101] A. E. Frankel , D. R. Fleming , B. L. Powell , R. Gartenhaus , Expert Opin. Biol. Ther. 2003, 3, 179. 12718740 10.1517/14712598.3.1.179

[102] R. Mazor , G. Kaplan , D. Park , Y. Jang , F. Lee , R. Kreitman , I. Pastan , Cell. Immunol. 2017, 313, 59. 28087047 10.1016/j.cellimm.2017.01.003 PMC5344129

[103] a) H. Wang , F. Franco , P.‐C. Ho , Trends Cancer 2017, 3, 583; 28780935 10.1016/j.trecan.2017.06.005

[104] a) Y. Ohue , H. Nishikawa , Cancer Sci. 2019, 110, 2080; 31102428 10.1111/cas.14069 PMC6609813

[105] a) J. Vent‐Schmidt , J. M. Han , K. G. MacDonald , M. K. Levings , Int. Rev. Immunol. 2014, 33, 110; 23947341 10.3109/08830185.2013.811657

[106] P. Kumar , A. Kumar , S. Parveen , J. R. Murphy , W. Bishai , Immunotherapy 2019, 11, 1117. 31361167 10.2217/imt-2019-0060 PMC7006781

[107] L. S. Cheung , J. Fu , P. Kumar , A. Kumar , M. E. Urbanowski , E. A. Ihms , S. Parveen , C. K. Bullen , G. J. Patrick , R. Harrison , Proc. Natl. Acad. Sci. U. S. A. 2019, 116, 3100. 30718426 10.1073/pnas.1815087116 PMC6386727

[108] P. Setlow , The Bacterial Spore: From Molecules To Systems (Eds: A. Driks , P. Eichenberger ), Wiley, New York 2016.

[109] R. A. Malmgren , C. C. Flanigan , Cancer Res. 1955, 15, 473. 13240693

[110] L. H. Dang , C. Bettegowda , D. L. Huso , K. W. Kinzler , B. Vogelstein , Proc. Natl. Acad. Sci. U. S. A. 2001, 98, 15155. 11724950 10.1073/pnas.251543698 PMC64999

[111] A. E. DeClue , S. M. Axiak‐Bechtel , Y. Zhang , S. Saha , L. Zhang , D. Tung , J. N. Bryan , Vet. Res. 2018, 49, 38. 29690928 10.1186/s13567-018-0531-0 PMC5937821

[112] J. T. Heap , J. Theys , M. Ehsaan , A. M. Kubiak , L. Dubois , K. Paesmans , L. Van Mellaert , R. Knox , S. A. Kuehne , P. Lambin , Oncotarget 2014, 5, 1761. 24732092 10.18632/oncotarget.1761 PMC4039107

[113] J. Nam , S. Son , K. S. Park , W. Zou , L. D. Shea , J. J. Moon , Nat. Rev. Mater. 2019, 4, 398.

[114] D. Nejman , I. Livyatan , G. Fuks , N. Gavert , Y. Zwang , L. T. Geller , A. Rotter‐Maskowitz , R. Weiser , G. Mallel , E. Gigi , Science 2020, 368, 973. 32467386 10.1126/science.aay9189 PMC7757858

[115] J. Stritzker , S. Weibel , P. J. Hill , T. A. Oelschlaeger , W. Goebel , A. A. Szalay , Int. J. Med. Microbiol. 2007, 297, 151. 17448724 10.1016/j.ijmm.2007.01.008

[116] a) T. Wang , N. Zheng , Q. Luo , L. Jiang , B. He , X. Yuan , L. Shen , Front. Immunol. 2019, 10, 1235;. 31214189 10.3389/fimmu.2019.01235 PMC6558076

